# Structural Characterization of the Millennial Antibacterial (Fluoro)Quinolones—Shaping the Fifth Generation

**DOI:** 10.3390/pharmaceutics13081289

**Published:** 2021-08-18

**Authors:** Aura Rusu, Ioana-Andreea Lungu, Octavia-Laura Moldovan, Corneliu Tanase, Gabriel Hancu

**Affiliations:** 1 Pharmaceutical and Therapeutical Chemistry Department, Faculty of Pharmacy, George Emil Palade University of Medicine, Pharmacy, Science, and Technology of Targu Mures, 540142 Targu Mures, Romania; aura.rusu@umfst.ro (A.R.); gabriel.hancu@umfst.ro (G.H.); 2 The Doctoral School of Medicine and Pharmacy, George Emil Palade University of Medicine, Pharmacy, Science, and Technology of Targu Mures, 540142 Targu Mures, Romania; ioana-andreea.lungu@umfst.ro (I.-A.L.); octavia.moldovan@umfst.ro (O.-L.M.); 3 Pharmaceutical Botany Department, Faculty of Pharmacy, George Emil Palade University of Medicine, Pharmacy, Science, and Technology of Targu Mures, 540142 Targu Mures, Romania

**Keywords:** fluoroquinolones, quinolones, structure-activity relationship, DNA gyrase, topoisomerase IV, antibacterial activity

## Abstract

The evolution of the class of antibacterial quinolones includes the introduction in therapy of highly successful compounds. Although many representatives were withdrawn due to severe adverse reactions, a few representatives have proven their therapeutical value over time. The classification of antibacterial quinolones into generations is a valuable tool for physicians, pharmacists, and researchers. In addition, the transition from one generation to another has brought new representatives with improved properties. In the last two decades, several representatives of antibacterial quinolones received approval for therapy. This review sets out to chronologically outline the group of approved antibacterial quinolones since 2000. Special attention is given to eight representatives: besifloxacin, delafoxacin, finafloxacin, lascufloxacin, nadifloxacin and levonadifloxacin, nemonoxacin, and zabofloxacin. These compounds have been characterized regarding physicochemical properties, formulations, antibacterial activity spectrum and advantageous structural characteristics related to antibacterial efficiency. At present these new compounds (with the exception of nadifloxacin) are reported differently, most often in the fourth generation and less frequently in a new generation (the fifth). Although these new compounds’ mechanism does not contain essential new elements, the question of shaping a new generation (the fifth) arises, based on higher potency and broad spectrum of activity, including resistant bacterial strains. The functional groups that ensured the biological activity, good pharmacokinetic properties and a safety profile were highlighted. In addition, these new representatives have a low risk of determining bacterial resistance. Several positive aspects are added to the fourth fluoroquinolones generation, characteristics that can be the basis of the fifth generation. Antibacterial quinolones class continues to acquire new compounds with antibacterial potential, among other effects. Numerous derivatives, hybrids or conjugates are currently in various stages of research.

## 1. Introduction

The historical moment of the emergence of a new class of antibacterial compounds was in 1945 when George Lesher and his team discovered the antimicrobial potential of 7-chloro-quinoline. This molecule was a compound with bactericidal action isolated during the synthesis and purification of chloroquine (antimalarial agent). Nalidixic acid, the first antibacterial quinolone (QN) derivative introduced in therapy, was discovered based on this compound (characterized by a naphthyridine nucleus) and was introduced into therapy in 1963 [1,2,3,4].

**Figure 1:**
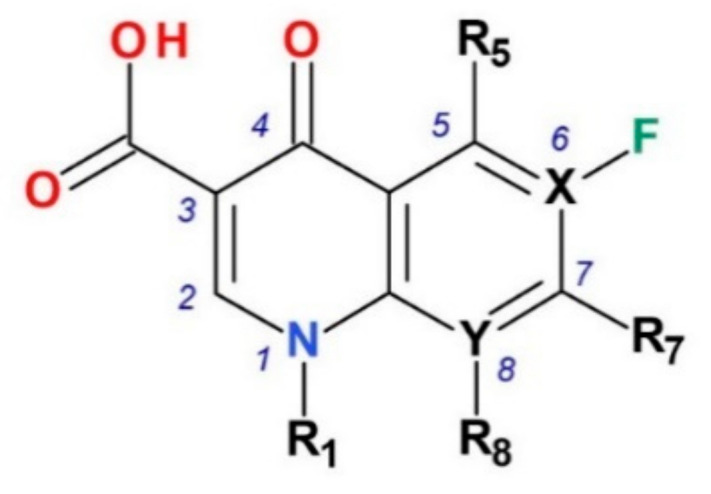
The general chemical structure of FQNs (1,4-quinolones) and numbering (X and Y = C or N).

The identification of a compound which is efficient against Gram-negative bacteria led to new derivatives as pipemidic acid, piromidic acid, oxolinic acid, cinoxacin and flumequine, the first generation of antibacterial quinolones (QNs) [5]. Flumequine was the first compound with a fluorine atom in the structure [6]. This optimization proved to be valuable for the next generation of antibacterial QNs. New quinoline derivatives were synthesized with superior pharmacokinetics and pharmacodynamic properties and a broader antibacterial spectrum [7,8]. Thus, the second generation of QNs was synthesized, obtained by introducing a fluorine atom in the sixth position of the quinolinic nucleus (Figure 1). These new QNs called generically “fluoroquinolones” (FQNs) presented an improved biological activity [9]. Numerous FQNs have been synthesized and studied. New compounds with an extended antibacterial spectrum, being active on both Gram-positive and Gram-negative bacteria (including *Pseudomonas aeruginosa*), have become valuable tools in therapy. The second generation comprises of both representatives for human use (norfloxacin, ciprofloxacin, ofloxacin), and for veterinary use (enrofloxacin) [3].

More valuable representatives were included in the third generation as levofloxacin (the *L*-enantiomer of ofloxacin) and gatifloxacin, which presented increased activity against Gram-positive bacteria (*Streptococcus* sp.), increased tissue penetration and half-life. Due to severe side effects (hypoglycemia), gatifloxacin is used only topically as eye drops [3,10,11]. Fourth generation FQNs have, in addition, acquired activity against anaerobic bacteria (e.g., moxifloxacin). Also, levofloxacin and moxifloxacin were included in the therapeutic protocols used in second-line multidrug-resistant tuberculosis [12,13]. The optimization of the chemical structure also led to a long half-life in moxifloxacin (13 h) [3,11]. Data on the discovery of antibacterial QN class representatives and their approval in therapy by the U.S. Food and Drug Administration (FDA) and/or European Medicines Agency (EMA) are briefly presented in Table 1.

The aim of this review is to present the progress in FQNs class since 2000. The newest FQNs introduced in therapy are highlighted and critically analyzed. Special attention is given to eight selected representatives (besifloxacin, delafloxacin, finafloxacin, lascufloxacin, levonadifloxacin, nadifloxacin, nemonoxacin, and zabofloxacin), approached from the perspective of physicochemical properties, antibacterial activity spectrum and advantageous structural modifications which influence for antibacterial efficiency.

## 2. Research Methodology

The literature research was conducted mainly on Clarivate Analytics and ScienceDirect databases using relevant keywords: (a) topic “fluoroquinolones”, “quinolones”, “antibacterials”; (b) title: “besifloxacin”, “delafloxacin”, “finafloxacin”, “lascufloxacin”, “nadifloxacin”, “levonadifloxacin”, “nemonoxacin”, “zabofloxacin”, and other relevant representatives of the FQNs class.

The articles were selected if they included relevant data regarding the aspects referred to in our review: discovery of the compound and the entities involved, data on approval in therapy, pharmaceutical formulations, infections treated by the targeted representatives, antibacterial activity spectrum, physicochemical properties, structure-activity relationships, elements of the safety profile related to chemical structure optimizations, bacterial resistance, and new QN derivatives. The manuscript contains relevant references, including those published in the first part of 2021.

Biovia Draw 2019 was used for drawing chemical structures (https://discover.3ds.com/biovia-draw-academic, accessed on 6 July 2021) [60]. MarvinSketch was used for drawing, displaying and characterizing chemical structures MarvinSketch 20.20.0, ChemAxon (https://www.chemaxon.com, accessed on 11 June 2021) [61].

## 3. Mechanism of Action

**Figure 2:**
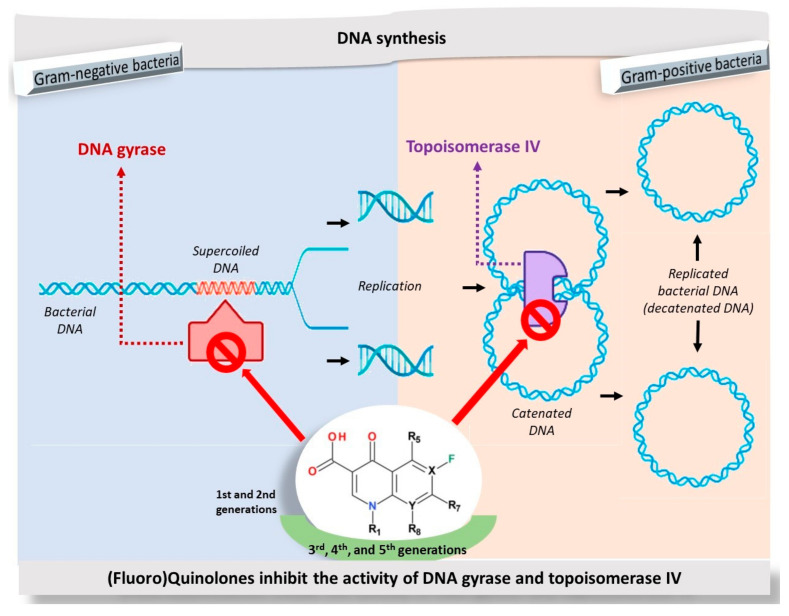
The mechanism of action of antibacterial (fluoro)quinolones.

The FQNs mechanism of action is well known and described in the literature [62,63,64,65,66]. It is known that FQNs act on two bacterial DNA enzymes: gyrase and topoisomerase IV (Figure 2) [67]. Thus, due to the covalent enzyme-DNA complex stabilization, DNA is cleaved. After this interaction, depending on the concentration, the death of the bacterial cell occurs in two ways: (1) at low concentration by blocking replication and transcription [62,68] and (2) at higher concentration (over the minimum inhibitory concentration) when the DNA topoisomerase is dissociated/removed [69], the DNA strands remain free, which leads to the chromosome fragmentation [70,71,72]. The advantage of new representatives is the action on both target enzymes and broadening the spectrum of activity against several types of pathogens [73,74]. In general, DNA gyrase from Gram-negative bacteria is more susceptible to inhibition than topoisomerase IV. On the other hand, topoisomerase IV from Gram-positive bacteria is more susceptible to inhibition than DNA gyrase [75].

Some studies have shown a correlation between FQNs lethality and reactive oxygen species (ROS) formation [76,77,78,79]. On the other hand, some issues about how do FQNs induce ROS accumulation remain unclear [80]. For example, Rodríguez-Rosado et al. (2018) studied the mechanisms of FQN-induced mutagenesis and the role of N-acetylcysteine in FQNs therapy to inhibit FQN-induced mutagenesis [81].

## 4. Classification into Generations of FQNs Used in Therapy

The most widely used classification of FQNs is the classification into generations based on of antibacterial activity and therapeutic use (Table 2).

## 5. Compounds in Therapy Since 2000

**Figure 3:**
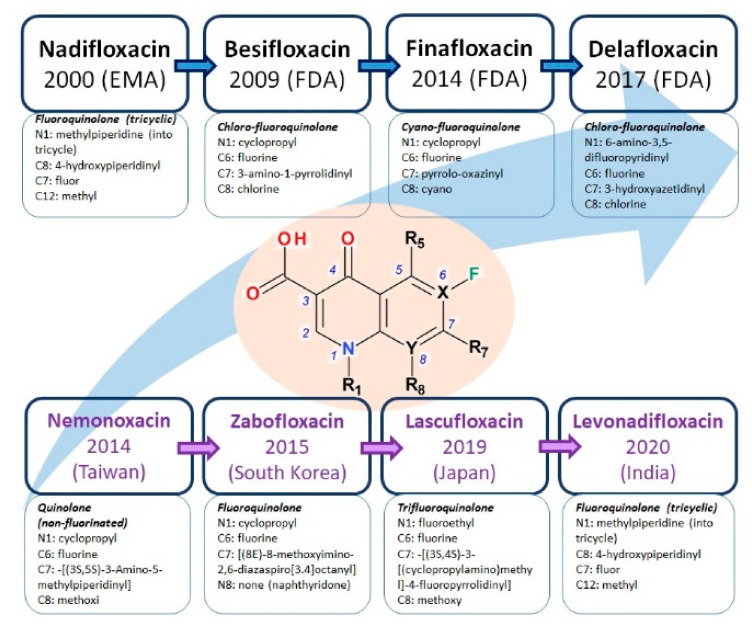
New FQNs chronology in therapy (since 2000) and essential structural characteristics.

The class of FQNs has evolved significantly since 2000, acquiring valuable representatives for therapy, with a low risk of occurrence of antibacterial resistance (Figure 3). The fourth-generation antibacterial QNs are very active on the DNA gyrase and topoisomerase IV, enzymes involved in bacterial DNA replication, transcription, repair and recombination. Recently approved by FDA or EMA are besifloxacin (2009), finafloxacin (2014) and delafloxacin (2017). The action on the two target enzymes confers the advantage of being effective on bacteria resistant to FQNs from previous generations; the development of bacterial resistance to the fourth generation representatives with multi-target properties is more difficult [42].

**Figure 4:**
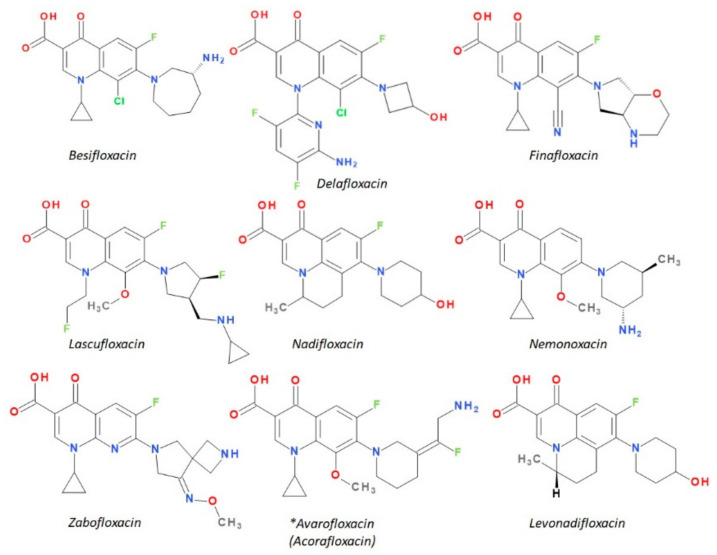
Chemical structures of newer approved antibacterial QNs (*final stage of approval).

The newest antibacterial 1,4-QNs used in therapy have diverse structural characteristics (Figure 4). According to the chemical structure of the base nucleus, these new compounds have a QN nucleus (besifloxacin, delafloxacin, finafloxacin, and nemonoxacin), a tricyclic ring including a QN nucleus (nadifloxacin), and naphthyridine nucleus (zabofloxacin). Regarding the presence of halogen atoms in the chemical structure, these compounds contain one fluorine atom (nadifloxacin and levonadifloxacin, finafloxacin, and zabofloxacin), one fluorine and one chlorine atom (besifloxacin), three fluorine atoms (lascufloxacin), three fluorine and one chlorine atoms (delafloxacin), but there is also an exception without any halogen atom (nemonoxacin).

Other FQNs from different generations that will be referred to for similarity to specific structural fragments, antibacterial activity or safety profile are described structurally in Figure S1—Supplementary Materials.

### 5.1. Besifloxacin

Besifloxacin is a chloro-FQN included in the fourth generation [101]. This new antibacterial molecule was developed for ophthalmic use by the SS Pharmaceutical SSP Co.Ltd. from Tokyo, Japan (former SS734). It has been approved by the FDA in 2009 and registered under the trade name Besivance (Bausch & Lomb Inc., Rochester, NY, USA) [46,98,101,102]. Besifloxacin is indicated for the treatment of bacterial conjunctivitis [98,103].

Besifloxacin’s spectrum of activity includes various bacterial species (broad-spectrum) [104,105,106] described in Table S1—Supplementary Materials. As for its mechanism of action, besifloxacin inhibits the two target enzymes, DNA gyrase and topoisomerase IV, essential in DNA replication [107]. Physicochemical properties of besifloxacin are comprised in Table 3. Exclusive topical administration is a peculiarity in the class of FQNs [101]. Most ophthalmic FQNs are also systemically (e.g., ciprofloxacin, ofloxacin, levofloxacin, moxifloxacin) adminstered. Gatifloxacin is administered for ophthalmic use only after the withdrawal from systemic use due to its side effects (hypo- and hyperglycemia) [43,108,109]. The approved pharmaceutical formulation of besifloxacin is an ophthalmic suspension 0.6%, which contains 6.63 mg of besifloxacin hydrochloride (equivalent to 6 mg of besifloxacin) [46,95,96,97,107]. At present, attempts are being made to develop several ophthalmic pharmaceutical formulas with besifloxacin, such as nanoemulsions [110], positively charged liposomes [111], and for the treatment of bacterial keratitis new loaded nanofibrous ocular inserts [112].

**Figure 5:**
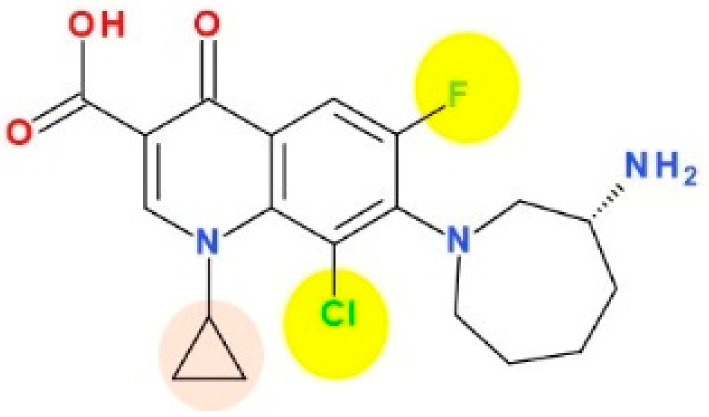
Chemical structure of besifloxacin.

Substitution at the N1 position in the FQN structure is essential for its antimicrobial activity. The N1 position substituent has been shown to control bacterial activity (potency) and some pharmacokinetic properties, like the increased volume of distribution and bioavailability [3]. That is why this substituent is common with other valuable FQNs such as ciprofloxacin and moxifloxacin (Figure S1—Supplementary Materials) [6,113,114]. It was considered that the cyclopropyl moiety from the N1 position of the QN nucleus confers besifloxacin activity against aerobic bacteria (Figure 5) [115].

**Table 3:** Physicochemical properties of besifloxacin.

Properties	Besifloxacin	Besifloxacin Hydrochloride	Ref.
Chemical name (IUPAC)	{7-[(3*R*)-3-aminohexahydro-1H-azepin-1-yl]-8- chloro-1-clyclopropyl-6-fluoro-1,4-dihydro-4-oxo-3-quinolinecarboxylic acid}	(+)-7-[(3*R*)-3-aminohexahydro-1H-azepin-1-yl]-8-chloro-1-cyclopropyl-6-fluoro-4-oxo-1,4-dihydroquinoline-3-carboxylic acid hydrochloride	[46] [97]
Chemical formula	C_19_H_21_ClFN_3_O_3_	C_19_H_21_ClFN_3_O_3_·HCl	[97]
Molecular weight	393.8 g/mol	430.40 g/mol	[116] [117]
Aspect	Not available	White to pale yellowish-white powder; white to light brown	[97,117,118]
Solubility	Insoluble in water 0.143 mg/mL (predicted values)	2 mg/mL (DMSO ^1^)	[46,106,116,118]
LogP	0.7; 0.54	Not available	[106]
p*K*a	6.0–7.0; 5.64—strongest acidic, 9.67—strongest basic (predicted values); 5.45 (carboxyl), 9.84 (amino) (calculated)	Not available	[46,61,106,116]
Melting point	Not available	Over 210 °C; 270.04 °C	[117,119]
Storage	Not available	At refrigerator; −20 °C	[117,118]

^1^ DMSO—Dimethyl sulfoxide.

Regarding the relationship between chemical structure and biological activity, substitution with a halogen (fluorine or chlorine) leads to decreased solubility, increased lipophilicity and increased penetration of the drug through cell membranes [120,121]. The electronic effects (inductive electron-attracting properties) are maximal for chlorine and very weak for fluorine [122]. The introduction of a fluorine atom at position C6 led to a spectacular increase in antimicrobial activity comparative to non-fluorinated QNs from the first generation. One fluorine atom in position C6 increased the degree of penetration into the bacterial cell and, at the same time, the activity against Gram-negative bacteria [6,113,114]. The fluorine atom appears to be essential in the mechanism of action.

A second substitution in the C8 position with a chlorine atom add an increased antimicrobial potency through the action on the target enzymes DNA gyrase and topoisomerase IV [46,123]. Also, C8 chlorine increases the antibacterial activity against FQN-resistant mutants of *Mycobacterium smegmatis* and *Staphylococcus aureus* [121].

Representatives of the second generation (ciprofloxacin, norfloxacin) with a piperazinyl group in the C7 position (Figure S1—Supplementary Materials) exhibit antibacterial activity against Gram-negative bacteria [6]. The C7 amino ring is a key substituent related to toxicity and solubility for analogues as clinafloxacin (with a 3-amino-1-pyrrolidinyl substituent) and sitafloxacin (with a (7*S*)-7-amino-5-azaspiro[2.4]heptan-5-yl) (Figure S1—Supplementary Materials). Unfortunately, clinafloxacin presented some side effects as phototoxicity and hypoglycemia. In addition, the solubility of clinafloxacin is poor and has inadequate stability in an aqueous solution [124]. Besifloxacin is administered only topically without systemic adverse reactions, being similar in terms of solubility. Assessment of besifloxacin toxicity conducted in silico presented a mutagenicity alert for two degradation products [125]. The replacement of the traditional piperazinyl group of the second generation with a hexahydro-1H-azepine cycle led to broadening the spectrum of activity on Gram-positive bacteria. The 3-aminohexahydro-1H-azepine ring contributes to specific action on the target enzyme DNA-gyrase, besifloxacin being superior to other FQNs in terms of antibacterial activity [126,127].

### 5.2. Delafloxacin

Delafloxacin is a recently approved FQN with an anionic chemical structure, from the fourth generation [38,128]. This new antibacterial molecule was developed for systemically use, both for oral and intravenous administration [39] by the Abbott Laboratories, Wakunaga Pharmaceutical (as ABT-492 compound or WQ-3034) and Melinta Therapeutics (former Rib-X Pharmaceuticals). It has been approved by the FDA in 2017 and registered under the trade name Baxdela for the treatment of acute bacterial skin and skin structure infections [39,129,130]. Physicochemical properties of delafloxacin are comprised in Table 4. The new product has the advantage of both oral and intravenous administration [131]. The parenteral form contains 433 mg delafloxacin meglumine (equivalent to 300 mg of delafloxacin) while the oral tablets contain 649 mg delafloxacin meglumine (equivalent to 300 mg of delafloxacin) [94]. Meglumine (1-deoxy-1-(methylamino)-D-glucitol) is a counterion used to increase the solubility of delafloxacin [132,133].

As a mechanism of action, delafloxacin inhibits the target enzymes DNA gyrase and topoisomerase IV, having a similar affinity for both [39,41]. An increased activity at acidic pH is an essential characteristic of this new chloro-FQN. Delafloxacin presents a broad spectrum of activity being active against both Gram-positive and Gram-negative bacteria, including methicillin-resistant *Staphylococcus aureus* (MRSA) and *Pseudomonas aeruginosa* (Table S1—Supplementary Materials) [130,134].

New formulations are being created to increase the effectiveness of delafloxacin. Optimized delafloxacin-loaded stearic acid (lipid) chitosan (polymer) hybrid nanoparticles proved to be superior comparative to delafloxacin standard suspension [135].

**Table 4:** Physicochemical properties of delafloxacin.

Delafloxacin	Properties	Ref.
Chemical name (IUPAC)	1-(6-amino-3,5-difluoropyridin-2-yl)-8-chloro-6-fluoro-7-(3-hydroxyazetidin-1-yl)-4-oxo-1,4-dihydroquinoline-3-carboxylic acid	[38,130]
Chemical formula	C_18_H_12_ClF_3_N_4_O_4_	[38,130]
Molecular weight	440.8	[38]
Aspect	Powder, white to beige	[136]
Solubility	0.0699 mg/mL (in water); 20 mg/mL (in DMSO ^1^)	[136,137]
LogP	1.67 (predicted value)	[136,137]
p*K*a	5.4; 5.62 (strongest acidic), −1.3 (strongest basic); 5.43 (carboxyl), −1.33 (piperidinic nitrogen atom), 14.77(hydroxyl)	[61,131,137]
Melting point	249.32 °C	[135,138]
Storage	Refrigerator: 2–8 °C	[136]

^1^ DMSO—Dimethyl sulfoxide.

Delafloxacin differs from other FQNs by the substituent 3-hydroxyazetidinyl at the C7 position. Also, in the N1 position, delafloxacin has an unusual 6-amino-3,5-difluoropyridinyl moiety that substantially enlarges the molecule’s molecular surface. This group is responsible for activity against Gram-positive bacteria [131,139]. This unique 3-hydroxyazetidinyl moiety on the C7 position confers acidic properties, and consequently, delafloxacin behaves as a weak acid (a non-zwitterion molecule with p*K*a 5.4) [131,139].

**Figure 6:**
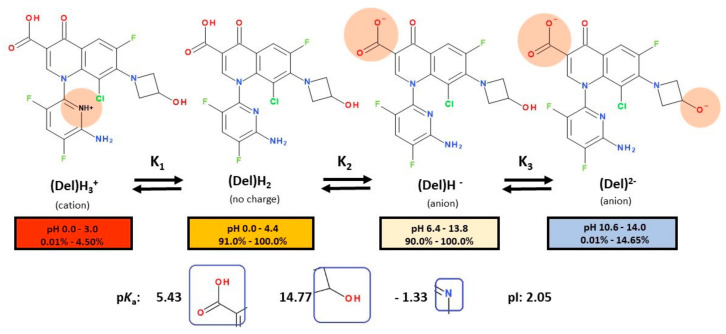
The macroprotonation scheme of delafloxacin and the step-wise protonation constants K_1_, K_2_ and K_3_. The carboxylate, hydroxyl, and the pyridine ring’s nitrogen atom (N1 position) are the most acidic, respectively, the most basic functions. All data were calculated with the MarvinSketch 20.20.0 version from ChemAxon [61].

At acidic pH, delafloxacin is an uncharged molecule, which is favorable for its passage through biological membranes (Figure 6).

These properties give delafloxacin increased activity in an acidic pH environment with decreased minimal inhibitory concentrations (MIC). Intracellularly, at neutral pH delafloxacin will be ionized into the anionic form and thus remain inside the pathogen agent [140]. So, this drug is beneficial against abscesses produced in the infection with *Staphylococcus aureus* [131,140].

In the C8 position, delafloxacin presents a chlorine substituent with an electron-withdrawing effect on the aromatic fragment of the QN nucleus (Figure 6), like besifloxacin (Figure 5). The chlorine substituent stabilizes delafloxacin molecule and could have a role in the reduction of the development of bacterial resistance. Thus, the whole polar molecule has increased activity [131,139]. C7 and C8 substitutions influence potency and spectrum of activity; both substitutions provide activity against anaerobic bacteria [139]. As a consequence, delafloxacin has proved activity against Gram-positive bacteria, especially against MRSA [139,141].

In the history of the development of FQNs, several trifluorinated molecules (e.g., fleroxacin, temafloxacin, trovafloxacin; Figure S1—Supplementary Materials) have been withdrawn due to severe side effects (Table 1). Fleroxacin (with N1-fluoroethyl, C6-fluor, C8-fluor) was the first promising trifluorinated representative but it was withdrawn due to severe phototoxic reactions [142]. Also, temafloxacin (with N1-difluorophenil) has been withdrawn due to severe hemolysis [19]. Finally, trovafloxacin (with N1-difluorophenil) has been withdrawn due to hepatotoxicity [142]. Unlike temafloxacin and trovafloxacin, delafloxacin contains a 6-amino-3,5-difluoropyridinyl substituent. This substituent appears to be more advantageous in reducing possible adverse reactions that have led to the withdrawal of the other trifluorinated compounds. However, the effects imprinted by the other substituents and the type of base nucleus must also be considered.

### 5.3. Finafloxacin

Finafloxacin (BAY35-3377) is a recent cyano-FQN included in the fourth generation. This new antibacterial molecule was developed by Bayer HealthCare Pharmaceuticals, Byk Gulden and MerLion Pharmaceuticals [42,50,99,143]. Relevant physicochemical properties are listed in Table 5. The FDA approved an otic suspension in 2014 and registered under the trade name Xtoro (developed by Novartis’s division, Alcon, Geneva, Switzerland). Finafloxacin is indicated for the treatment of acute otitis externa [42,99].

At the same time, finafloxacin is in various stages of clinical trials to evaluate the efficacy of oral and intravenous formulations. These forms are intended for the treatment of uncomplicated and complicated urinary tract infections, pyelonephritis and *Helicobacter pylori* infections [99,144,145,146,147]. Finafloxacin has demonstrated broad-spectrum activity against a range of pathogens [148]. This cyano-FQN is active both in vitro and in vivo against *Pseudomonas aeruginosa* and *Staphylococcus aureus* [99].

As for the mechanism of action, similar to fourth-generation representatives, finafloxacin has a high affinity for the two target enzymes, DNA-gyrase and topoisomerase IV [99]. Antimicrobial activity of finafloxacin is enhanced in acidic conditions (pH 5.8) against multiple pathogens, including skin and urinary pathogens. Finafloxacin exhibits activity at neutral pH comparable to previous generations of FQNs. Also, a more prolonged post-antibacterial effect against multiple species was observed compared to other FQNs at acidic pH. The development of bacterial resistance to finafloxacin is less likely in acidic conditions [99,100,149,150].

**Table 5:** Physicochemical properties of finafloxacin.

Properties	Finafloxacin	References
Chemical name (IUPAC)	(-)-8-Cyano-1-cyclopropyl-6-fluoro-7-((4a*S*,7a*S*)-hexahydropyrrolo(3,4-b)-1,4-oxazin-6(2*H*)-yl) -4-oxo-1,4-dihydroquinoline-3-carboxylic acid	[49,99,151]
Chemical formula	C_20_H_19_FN_4_O_4_	[49]
Molecular weight	398.4	[49]
Aspect	Powder, white to beige; white to yellowish (hydrochloride salt);	[152,153]
Solubility	0.208 mg/mL (in water) 2 mg/mL (in DMSO ^1^) 5.5 mg/mL (in water, hydrochloride salt)	[151,152,153]
LogP	−0.5; −1.1 (predicted)	[151]
p*K*a	5.6 (carboxylate) and 7.8 (nitrogen at C7)	[143,153]
Melting point	Not available	
Storage	−20 °C	[152]

^1^ DMSO—Dimethyl sulfoxide.

**Figure 7:**
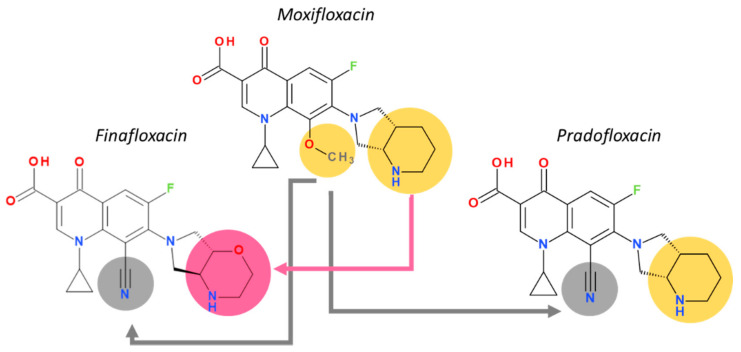
Relevant structural elements to the antibacterial activity of moxifloxacin (C7—pyrrolo-piperidinyl, C8—methoxi), pradofloxacin (C7—pyrrolo-piperidinyl, C8—cyano) and finafloxacin (C7—pyrrolo-oxazinyl, C8—cyano).

The molecule optimizations include a pyrrolo-oxazinyl moiety at the C7 position and a cyano-substituent at the C8 position (Figure 7). Bearing a zwitterion chemical structure (carboxylate at C3 position and pyrrolo-oxazinyl at C7 position) finafloxacin presents two dissociation constants (Table 5) [143]. The voluminous C7 substituent confers to the molecule’s unique characteristic of not being recognized by efflux transporters, the key to decreased bacterial resistance development [48]. The C7 pyrrolo-oxazinyl fragment emerged from the C7 azabicycle (pyrrolidine-piperidine) fragment of moxifloxacin and pradofloxacin (Figure 7), which confers the ability to remain longer in the bacterial cell (difficult to efflux molecules) [154,155].

A cyano-substituent at C8 is also present in the chemical structure of pradofloxacin, a veterinary-approved FQN classified in the third generation. This compound can be considered an analogue of moxifloxacin (fourth generation) due to the methoxy group at the C8 position which has been replaced by a cyano group. Pradofloxacin is more active against Gram-positive bacteria comparative to previous generations. Also, pradofloxacin exhibits good activity against anaerobic bacteria, similar to moxifloxacin, and an equal or lower activity against Gram-negative bacteria [36,156,157]. The C8 cyano group appears to play an essential role in activity against Gram-positive when comparing finafloxacin with pradofloxacin (Figure 7).

At the N1 position, finafloxacin has a cyclopropyl substituent similar to second-generation ciprofloxacin and fourth-generation besifloxacin.

Finafloxacin and delafloxacin in acidic conditions (pH 5.0–6.0 and respectively pH ≤ 5.5) are more active than other FQNs, but for different reasons. In the key C7 position of delafloxacin, the 3-hydroxyazetidine without a basic group is the substituent that confers acidic properties. In finafloxacin, the nitrogen atom from the oxazine fragment is responsible for the great activity in acidic conditions (can accept protons) [42,61]. In acidic conditions, finafloxacin is very active against *Staphylococcus aureus* due to an increased uptake in the bacteria [48].

### 5.4. Lascufloxacin

**Figure 8:**
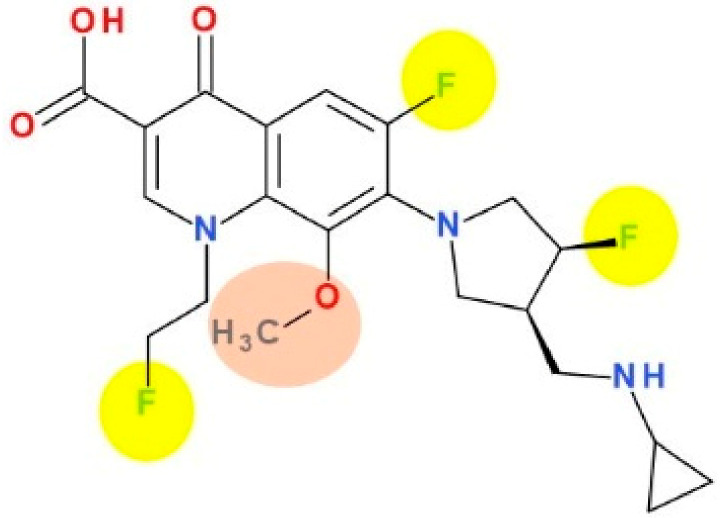
Chemical structure of lascufloxacin.

Lascufloxacin (KRP-AM1977) is a new FQN (Figure 8) developed in Japan by Kyorin Pharmaceutical Co., Ltd. [158]. Some physicochemical properties of lascufloxacin are comprised in Table 6.

**Table 6:** Physicochemical properties of lascufloxacin.

Lascufloxacin	Properties	References
Chemical name (IUPAC)	7-[(3*S*,4*S*)-3-[(cyclopropylamino)methyl]-4-fluoropyrrolidin-1-yl]-6-fluoro-1-(2-fluoroethyl)-8-methoxy-4-oxoquinoline-3-carboxylic acid	[159]
Chemical formula	C_21_H_24_F_3_N_3_O_4_	[159]
Molecular weight	439.4 g/mol	[159]
Aspect	White to off-white solid powder	[160]
Solubility	Slightly soluble in chloroform, very slightly soluble in DMSO ^1^, insoluble in water	[160,161]
LogP	1.79 (nonionic species)	[61]
p*K*a	5.64 (carboxyl), 9.75 (secondary amino group)—calculated	[61]
Melting point	Not available	
Storage	0–4 °C (short term, days to weeks); −20 °C (long term, months), dry and dark conditions	[162]

^1^ DMSO—Dimethyl sulfoxide.

This new antibacterial agent was approved recently in Japan (2019) as a hydrochloride salt (oral formulation, Lasvic^®^ 75 mg tablets) for the treatment of respiratory infections (including community-acquired pneumonia (CAP)) and ear, nose and throat infections [163,164]. Lascufloxacin acts by binding to the target enzymes, DNA gyrase and topoisomerase IV (inhibiting DNA synthesis), similar to other antibacterial FQNs [165]. Also, lascufloxacin demonstrated a high binding capacity to phosphatidylserine (a component of human cell membranes; primary surfactant of alveolar epithelial fluid). Lascufloxacin is superior in tissue penetration (head and neck infections) compared with levofloxacin, garenoxacin, and moxifloxacin [158].

Lascufloxacin proved to be very active against Gram-positive bacteria, including resistant species (Table S1—Supplementary Materials). Also, lascufloxacin is very promising against FQN-resistant pathogens located in the respiratory tract [166,167,168]. For example, a potent activity of lascufloxacin was proved against first-step mutants of *Streptococcus pneumoniae*. Being a new FQN, lascufloxacin has a significant potential to fight against the installation of bacterial resistance in pneumococcal infections [169].

Another formulation for parenteral administration (KRPAM1977Y) was recently approved in Japan (November 2020) [56,163,164]. Lasvic^®^ is the generic brand and contains lascufloxacin hydrochloride 150 mg [163]. A phase I clinical study of lascufloxacin was recently performed in Japan. The pharmacokinetic and safety profile was assessed in non-elderly healthy men comparative to elderly healthy men. The obtained results proved that lascufloxacin has a safe pharmacokinetic profile without dose adjustments for the two groups of men [170]. The average half-life of lascufloxacin is about 16.1 h after 100 mg (orally administered) [168]. This new FQN presented an extensive distribution into the lungs [171].

The substitution with a fluoroethyl of the N1 position is similar to the fleroxacin, the first trifluorinated antibacterial QN, whose use has been limited by the severe phototoxicity [142]. The fluoroethyl substituent was correlated with the photosensitising effect [172,173]. However, according to the data published so far lascufloxacin has a good safety profile [170].

In the C7 position, FQNs usually have nitrogen heterocycles (five or six atoms), aminopyrrolidines and piperazines. In lascufloxacin chemical structure, an unusual structural fragment is present in the C7 position, a main pyrrolidine heterocycle substituted with a (cyclopropylamino)methyl moiety. This position is essential for interaction with DNA gyrase or topoisomerase IV [114,174,175]. Also, an aminopyrrolidine improves Gram-positive activity, proven by the clinafloxacin representative [176]. Thus, clinafloxacin (with a C8 chlorine atom) was associated with severe side effects (phototoxicity hypoglycaemia) [18]. Sitafloxacin, a FQN approved in Japan (2008) and Thailand (2012) contains in the C7 position a pyrrolidinyl fragment included in a spiro substituent, [(7S)-7-amino-5-azaspiro[2.4]heptanyl] [57,177]. This FQN produces mild to moderate adverse reactions (mostly gastrointestinal disorders and laboratory abnormalities, phototoxicity potential) [178,179]. Another pyrrolidinyl fragment is found in the structure of zabofloxacin, also included in a spiro substituent, [(8*Z*)-8-methoxyimino-2,6-diazaspiro[3.4]octanyl] (chapter 5.7). Unlike sitafloxacin, zabofloxacin is considered a well-tolerated FQN with acceptable side effects [143].

The methoxy substituent in the C8 position improves activity and enhances antimicrobial potency, especially against anaerobic bacteria [158], similar to moxifloxacin and pradofloxacin [34,36,114].

### 5.5. Nadifloxacin and Levonadifloxacin

Nadifloxacin is the first FQN approved for dermatological use, being classified in the second generation. This new antibacterial molecule was developed by the Otsuka Pharmaceuticals from Japan (former OPC-7251) [27]. The physicochemical properties of nadifloxacin are comprised in Table 7. It has been approved in Japan in 1993 (Aqutim) and in several countries in the European Union (2000). Nadifloxacin was initially approved for the treatment of acne vulgaris, and then for other skin infections (1998) [26,89,180,181]. The approved topical formulation is a cream containing 1% nadifloxacin [89,180].

Nadifloxacin proved to be effective against Gram-positive (including MRSA and coagulase-negative staphylococci), aerobic Gram-negative, and anaerobic bacteria (Table S1—Supplementary Materials) [88]. Superior antibacterial activity of nadifloxacin has been reported comparative with ciprofloxacin, clindamycin and erythromycin against *Propionibacterium acnes, Staphylococcus epidermidis*, methicillin-susceptible *Staphylococcus aureus* (MSSA), and MRSA. Moreover, nadifloxacin did not have an additional effect on resistance [182].

Regarding the mechanism of action, nadifloxacin inhibits the enzyme DNA gyrase, involved in the synthesis and replication of bacterial DNA [180,183]. Also, nadifloxacin proved to have inhibitory effects upon activated T cells and keratinocytes, as a part of the mechanism involved in its effect against inflammatory acne [87].

**Table 7:** Physicochemical properties of nadifloxacin.

Nadifloxacin	Properties	References
Chemical name (IUPAC)	7-fluoro-8-(4-hydroxypiperidin-1-yl)-12-methyl-4-oxo-1-azatricyclo[7.3.1.0⁵,¹³]trideca-2,5,7,9(13)-tetraene-3-carboxylic acid	[184]
Chemical formula	C_19_H_21_FN_2_O_4_	[184]
Molecular weight	360.4 g/mol	[184]
Aspect	White; light yellow powder	[185,186]
Solubility	25 mg/mL (in DMF ^1^), 20 mg/mL (in DMSO ^2^), 0.25 mg/mL (in ethanol), insoluble in water	[185,186,187,188]
LogP	2.47; 1.77 (calculated)	[61,187]
p*K*a	5.94 (carboxyl), 0.44 (piperidinic nitrogen atom), 15.18 (hydroxyl)	[61]
Melting point	245–247 °C (decomposition)	[87,189]
Storage	−20 °C	[188,189]

^1^ DMF—Dimethylformamide, ^2^ DMSO—Dimethyl sulfoxide.

**Figure 9:**
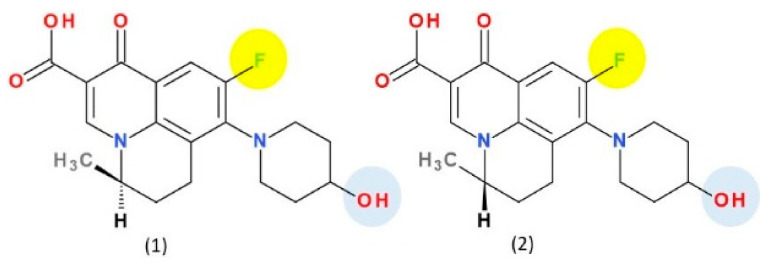
Chemical structures of nadifloxacin (**1**) and levonadifloxacin (**2**).

Nadifloxacin (Figure 9) is a tricyclic FQN very similar to ofloxacin (Figure S1—Supplementary Materials) [184,190]. The essential modification is the replacement in the C8 position of the methyl-piperazine moiety from the ofloxacin structure with a 4-hydroxypiperidine moiety. Nadifloxacin is considered a lipophilic compound compared to ofloxacin (logP = −0.39) [191,192]. In therapy, nadifloxacin is used as a racemic [26]. However, the two enantiomers have different biological activities. Thus, it is known that the levorotatory (*S*)-isomer is 64- to 256-times more potent than the (*R*)-isomer. Also, the levorotatory (*S*)-isomer is approximately twice as active as the racemate against Gram-positive and Gram-negative pathogens [180]. This stereoisomer is under study as an arginine salt for intravenous administration (WCK 771) and is known as levonadifloxacin [193]. In general, the introduction of hydroxyl groups into the structure of a compound will produce analogues with increased hydrophilicity and low solubility in lipids. The hydroxyl group into the chemical structure provides a new center for hydrogen bonding, which can influence the binding of the analogue to the active center of the target, the biological activity, and metabolism [120]. The introduction of a hydroxyl group on the piperidine heterocycle at position C8 confers a slight increase of the hydrophilic character of nadifloxacin and an increase of acidic properties. However, the molecule has a LogP 2.47, which denotes increased lipophilia and low aqueous solubility. This structural optimization is present in the structure of delafloxacin, but on an azetidine heterocycle on C7 position. Nevertheless, the LogP value of delafloxacin is lower (1.67—predicted value) than that of nadifloxacin.

Levonadifloxacin ((12*S*)-7-fluoro-8-(4-hydroxypiperidin-1-yl)-12-methyl-4-oxo-1-azatricyclo[7.3.1.05,13]trideca-2,5,7,9(13)-tetraene-3-carboxylic acid) is the active *S*(−) isomer of nadifloxacin recently approved in India (Figure 9) [194,195]. The *S*(−) isomer of nadifloxacin, has been shown to be more potent than the *R*(+) isomer and twice as active as the racemic form of nadifloxacin against Gram-positive and Gram-negative bacteria. It is a new broad-spectrum anti-MRSA agent belonging to the benzoquinolizine subclass of QN [180,195].

Levonadifloxacin (WCK 771) (*S*-(−)-9-fluoro-6,7-dihydro-8-(4-hydroxypiperidin-1-yl)-5-methyl-1-oxo-1H,5H-benzo[i,j] quinolizine-2-carboxylic acid *L*-arginine salt tetrahydrate) is administered parenterally (intravenous) in the form of an *L*-arginine salt while its prodrug alalevonadifloxacin (WCK 2349) ((*S*)-(−)-9-fluoro-8-(4-*L*-alaninyl oxypiperidin-1-yl)-5-methyl-6,7-dihydro-1-oxo-1H,5H-benzo[i,j] quinolizine-2-carboxylic acid, methane sulfonic acid salt) in the form developed of an *L*-alanine ester mesylate salt can be administered orally. Both substances are being developed by Wockhardt Limited (India) [193,196].

Both levonadifloxacin and alalevonadifloxacin have successfully completed phase II and phase III trials, indicating that they are clinically appealing therapeutic alternatives for infections caused by multidrug-resistant Gram-positive pathogens. Due to simultaneous inhibition of DNA gyrase and topoisomerase IV, both representatives exhibit significant antibacterial activity against Gram-negative and Gram-positive bacteria, with an emphasis on MRSA [193,197,198]. Levonadifloxacin has the advantage of being potent against resistant pathogens with a very low frequency of mutation [199,200]. Both substances have been studied for the treatment of acute skin and skin structure bacterial infections, community-acquired bacterial pneumonia, and other infections in both non-clinical and clinical studies [193,197,199].

Because of its non-basic hydroxy piperidine side chain, levonadifloxacin remains un-ionized at acidic pH, allowing it to enter the bacterial cell more easily. As a result, levonadifloxacin’s efficacy in acidic conditions increases significantly; this characteristic might be helpful for intracellular activity and antibacterial action [197]. Various in vitro and in vivo investigations have established levonadifloxacin’s antibacterial spectrum against Gram-positive, Gram-negative, atypical, and anaerobic pathogens [201].

The excellent bioavailability of oral formulations can be helpful in the smooth switch from parenteral to oral therapy. Both medication forms have well-established pharmacokinetics and safety; in the phase I trial, there were no notable severe or unfavourable clinical or laboratory side effects, indicating that both formulations are well tolerated [193].

### 5.6. Nemonoxacin

**Figure 10:**
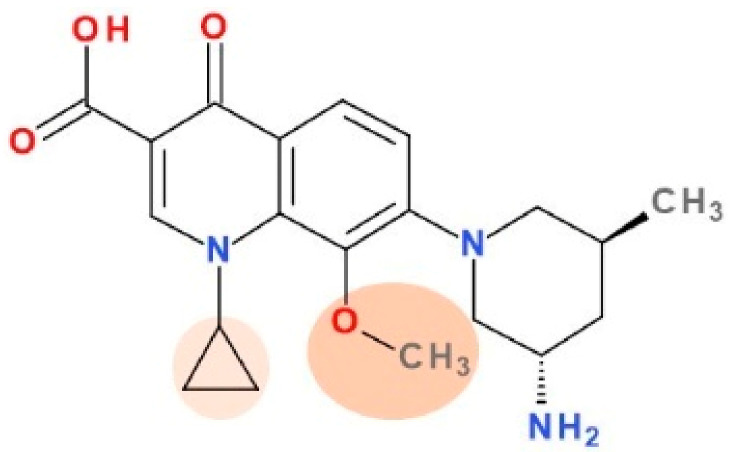
Chemical structure of nemonoxacin.

Nemonoxacin is a new non-fluorinated QN chemotherapeutic (Figure 10). Nemonoxacin (TG-873870) was developed by TaiGen Biotechnology under the commercial name of Taigexyn^®^ for the treatment of CAP, both orally and intravenously as well as the treatment of diabetic foot ulcer infections and skin and soft tissue infections [202]. Procter & Gamble initially developed nemonoxacin, and TaiGen Biotechnology was granted a worldwide license in October 2004. In March 2014, it gained its first global approval in Taiwan to treat CAP in adults. TaiGen Biotechnology holds the nemonoxacin patent portfolio, which protects the drug’s use, composition, and manufacturing techniques until 2029 [53,54].

A clinical study (phase II) regarding the safety and efficacy of nemonoxacin in diabetic foot infections was completed [203,204]. As a result, the FDA authorized oral administration of nemonoxacin to treat CAP and bacterial skin infections [166,205,206].

Taigexyn product contains nemonoxacin malate hemihydrate salt [207,208]. The understudy intravenously administered formula contains nemonoxacin malate sodium chloride [209]. Physicochemical properties of nemonoxacin are comprised in Table 8.

**Table 8:** Physico-chemical properties of nemonoxacin.

Nemonoxacin	Properties	References
Chemical name (IUPAC)	7-[(3*S*,5*S*)-3-Amino-5-methylpiperidin-1-yl]-1-cyclopropyl-8-methoxy-4-oxo-1,4-dihydroquinoline-3-carboxylic acid	[210,211]
Chemical formula	C_20_H_25_N_3_O_4_	[210,211]
Molecular weight	371.4 g/mol	[210,211]
Aspect	Not available	
Solubility	Insoluble in water 0.453 mg/mL (predicted values)	[212]
LogP	0.32; −0.44	[212]
pKa	5.73—strongest acidic, 9.66—strongest basic (predicted values); 5.53 (carboxyl), 0.28 (piperidinic atom nitrogen), 9.83 (amino)	[212] [61]
Melting point	Not available	
Storage	Not available	

The QN ring’s C8 methoxy substituent improves antibacterial efficacy against Gram-positive bacteria and lowers the selection of resistant variants. The fluorine substituent absence may reduce the frequency of dangerous side effects [143]. The addition of a methoxy group at position C8 allows nemonoxacin to target both DNA gyrase and topoisomerase IV, resulting in a broader spectrum of activity and less mutant selection [213,214].

Nemonoxacin is similar to gatifloxacin (a fourth-generation FQN) (Figure S1—Supplementary Materials), except for the lack of C6 substitutions of fluorine and the 5′-methyl piperidinyl ring at C7 position of the QN ring [215,216]. Gatifloxacin proved to be more active against *Streptococcus pneumoniae* than second generation ciprofloxacin or the third generation levofloxacin [34,216]. This increased activity against *Streptococcus pneumoniae* is similar to moxifloxacin, another fourth-generation FQN with a methoxy group at C8 [34]. The C8 methoxy group of nemonoxacin probably potentiates the same level of inhibition of DNA gyrase and topoisomerase IV in *Streptococcus pneumoniae* cells, and confers low mutant selectivity [216].

The introduction of a piperidine substituent to C7 has not been common in the past. Few representatives were obtained with a piperidine substituent in the C7 position. Among them is balofloxacin (Figure S1—Supplementary Materials), developed by Choongwae Pharma and approved only in Korea to treat urinary tract infections [32]. At the C7 position, balofloxacin exhibits a 3-(methylamino)piperidinyl moiety [217]. Balofloxacin did not have the expected success. This FQN from the third generation reported total adverse drug reaction rates of 5.4% compared to levofloxacin (1.3%). The side effects reported were gastrointestinal, CNS and skin-related [18,23,218]. Shankar et al. (2018) published the predicted toxicity of balofloxacin and its metabolites (*in silico* study); most of the metabolites are found to be immunotoxic [219]. Avarofloxacin (acorafloxacin, JNJ-Q2) is a new promising FQN in development with a piperidine substituent in the C7 (discussed in a later chapter) [143,220].

*In vitro* investigations have shown that nemonoxacin has broad-spectrum antibacterial activity, including activity against microorganisms resistant to other antibacterial drugs, including multidrug-resistant *Streptococcus pneumoniae* and MRSA [221,222]. It is used to treat Gram-positive and Gram-negative bacterial infections, including MSSA and MRSA, with once-daily oral and intravenous preparations. Nemonoxacin presented higher activity than levofloxacin and ciprofloxacin against a variety of Gram-positive bacteria, including resistant species. For Gram-negative bacteria such as *Escherichia coli, Hemophilus influenzae*, *Klebsiella pneumoniae*, and *Pseudomonas aeruginosa*, nemonoxacin activity was equivalent to levofloxacin and ciprofloxacin [223]. In vitro testing of nemonoxacin against 2440 clinical isolates revealed that it had better efficacy against most Gram-positive species than levofloxacin and moxifloxacin [224]. In the murine model of systemic, pulmonary, or ascending urinary tract infection, nemonoxacin outperforms the most commonly used FQNs [225]. Compared to other FQNs, nemoxacin has a low predisposition for generating resistant infections because bacteria develop resistance to nemonoxacin only when three distinct mutations happen in the QN resistance-determining region of the relevant gene [214].

Nemonoxacin has a favourable pharmacokinetic profile, being rapidly absorbed, having a high bioavailability, and a large distribution volume; it has a relatively long elimination half-life of more than 10 h and achieves maximum concentration (C_max_) 1–2 h after oral administration. Approximately 60–75% of the given dosage is excreted in an unaltered state; only a minor metabolite (5%) was identified due to metabolic processes [213,226]. Nemonoxacin is well tolerated, the gastrointestinal and neurological system-related are the most prevalent side effects of oral administration, with a frequency equivalent to that of levofloxacin therapy [227].

Nemonoxacin may play a significant role in the treatment of many infectious illnesses due to its equivalent or higher potency against Gram-positive bacteria and similar activity against Gram-negative pathogens when compared with other classic FQNs.

### 5.7. Zabofloxacin

Zabofloxacin is a FQN approved in 2015 only in South Korea [55,56]. The new compound (PB-101, DW224a, DW224aa) was developed by Dong Wha Pharm. Co. Ltd. (Seoul, Korea) [56]. There were two salts in development: DW224a as zabofloxacin hydrochloride, and DW224aa as zabofloxacin aspartate [228]. The physicochemical properties of zabofloxacin are comprised in Table 9. Zabofloxacin is marketed under the name Zabolante to treat acute bacterial exacerbation of chronic obstructive pulmonary disease by oral administration. Zabolante contains 512.98 mg zabofloxacin aspartate hydrate (equivalent to 366.69 mg of zabofloxacin) [56,229].

Zabofloxacin activity is mainly against Gram-negative and Gram-positive respiratory pathogens, especially against *Streptococcus pneumoniae*, and drug-resistant *Neisseria gonorrhoeae* (Table S1—Supplementary Materials) [230,231,232]. In Phase III clinical trial zabofloxacin (367 mg once daily; 5 days) proved to be as efficient as moxifloxacin (400 mg once daily; 7 days) in treating chronic obstructive pulmonary disease exacerbations [92].

Zabofloxacin mechanism of action is similar to other FQNs with a broad spectrum against respiratory pathogens [233].

**Table 9:** Physicochemical properties of zabofloxacin.

Zabofloxacin	Properties	References
Chemical name (IUPAC)	1-cyclopropyl-6-fluoro-7-[(8*E*)-8-methoxyimino-2,6-diazaspiro[3.4]octan-6-yl]-4-oxo-1,8-naphthyridine-3-carboxylic acid	[234]
Chemical formula	C_19_H_20_FN_5_O_4_	[234]
Molecular weight	401.4 g/mol	[234]
Aspect	Not available	
Solubility	0.196 mg/mL in water (calculated)	[235]
LogP	−0.89 (calculated); 2.43 for non ionic species	[61,235]
p*K*a	5.53—strongest acidic, 9.41—strongest basic; 5.34 (carboxyl), 2.66 (N8 atom), 0.30 (N imino), 9.50 (N6′ from spiro heterocycle) (calculated)	[61,235]
Melting point	155 °C	[236]
Storage	Not available	

**Figure 11:**
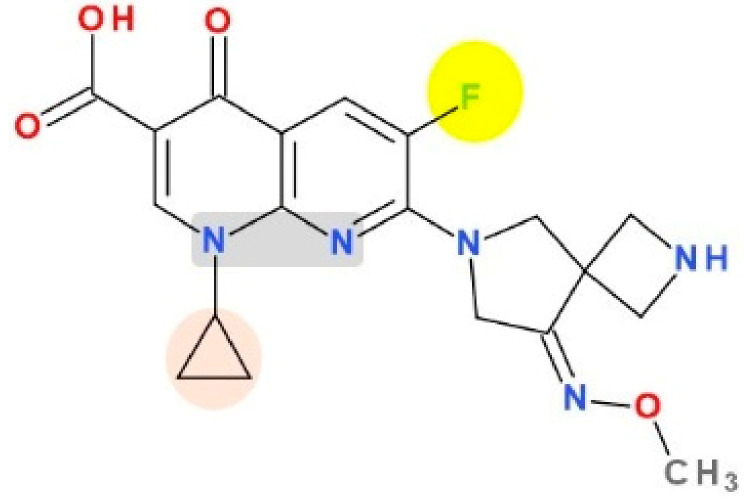
Chemical structure of zabofloxacin.

Unlike previous compounds in the class of new antibacterial QNs, zabofloxacin is a fluoronaphthyridone (Figure 11) [56].

At the C7 position, zabofloxacin has an unusual heterocycle, a spiro substituent (2,6-diazaspiro[3.4]octan) substituted in C8′ with an imino methoxy group. This new compound could be considered an analogue of gemifloxacin (from the fourth-generation) by optimizing the heterocycle from position C7 (Figure S1—Supplementary Materials) [34]. Although the antibacterial activity of gemifloxacin was superior to moxifloxacin, unfortunately, due to side effects (mainly rash), it was withdrawn [34,45]. Increasing the volume of the C7 heterocycle by maintaining the substituted methoxy-imino pyrrolidine ring resulted in a compound with acceptable side effects [228]. Various spiro compounds with antibacterial activity have been published [237,238,239,240].

## 6. Is the Fifth Generation of Antibacterial FQNs Outlined?

The therapeutic value of the newer FQNs discussed in this review is undeniable. This recent evolution in the class of FQNs is based on several essential elements of the chemical structure, which are further analyzed (Table 10).

The substituent at the N1 position increased potency, antibacterial activity and pharmacokinetic properties [241]. Cyclopropyl is known as the most potent optimization at the N1 position [113]. Thus, the substitution to N1 with cyclopropyl was preferred in four chemical structures of new FQNs (Table 10). An interesting issue is that in the past difluorophenyl in the N1 position was associated with several side effects (temafloxacin, trovafloxacin) [177]. This substitution is optimized in the chemical structure of delafloxacin with a 6-amino-3,5-difluoropyridinyl moiety. Basic groups are known to form salts in biological media. Substitution with basic groups will produce analogues with lower lipophilia and increased solubility in water. The more basic is the optimized molecule, the more likely it is to form salts and the less likely it is to be transported through a lipid membrane. The introduction of an amino group is likely to increase the binding of delafloxacin to target enzymes via hydrogen bonds [120]. The substituted pyridine residue proved to be more advantageous for the safety profile of delafloxacin comparative to older FQNs [134]. However, it should be noted that the incorporation of an aromatic amine (considered a toxophore) into the structure of a compound is avoided because aromatic amines are often highly toxic and carcinogenic [120].

The fluorine atom has an essential role in medicinal chemistry. Comparative to hydrogen, fluorine atom has small size, van der Waals radius of 1.47 Å versus van der Waals radius of 1.20 Å. In addition, the fluorine atom is highly electron-withdrawing (with impact on p*K*_a_), the C-F bond is more stable than the C-H bond, and the lipophilicity of the fluorinated molecule is higher than the non-fluorinated version. Also, substitution with a fluorine atom confers metabolic stability, influences the metabolic pathways and pharmacokinetic properties, increases the permeability of the molecule through cell membranes and the binding affinity to the target proteins [242,243]. Changes in potency produced by the introduction of a halogen-containing substituent or halogen group depend on the substitution position [120]. In the C6 position, the fluorine substituent increased the potency of FQNs versus non-fluorinated QNs. The fluorine atom increased the bacterial cell penetration and the affinity to the DNA-gyrase [113,241]. Over time, most synthesized compounds retain fluoride substitution at C6. All new compounds discussed in this review have a fluorine atom in position 6 (respectively 7 for nadifloxacin), except nemonoxacin. The other structural optimizations in the case of nemonoxacin compensated for the effect that the fluorine atom would have brought.

The substituent from the C7 position increased potency, the spectrum of antibacterial activity, safety profile, and pharmacokinetic properties. This position on the QN nucleus was most often targeted for structural optimizations. Advantageous optimizations for antibacterial activity were a five or six-membered nitrogen heterocycle, four-membered heterocycle, piperazinyl, fluorine or chlorine atoms, substituted hydrazine fragment or bicyclic substitution [241].

A C7 pyrrolidine substituent increases the activity against Gram-positive bacteria. An attempt to optimize the structure of FQNs with a C7 pyrrolidine substituent was clinafloxacin [244,245]. Although it had potential antibacterial clinafloxacin was associated with phototoxicity and hypoglycaemia [18].

Lascufloxacin contains an optimized pyrrolidine nucleus which confers great potential for treating respiratory infections (including CAP) and ear, nose and throat infections [158,246]. Regarding the new compounds, the pyrrolidine nucleus is found condensed with another heterocycle (morpholine) in finafloxacin’s chemical structure. In zabofloxacin’s chemical structure, the pyrrolidine nucleus is part of a spiro fragment.

A potential increase of antibacterial activity may appear with C7 and C8 cyclization. C8 substituents are essential for target affinity, because of the planar configuration of the molecule. Fluorine or chlorine, methyl or methoxy substituents proved to enhance antibacterial potency [247]. Out of these, the methoxy substituent is found in the structure of the representatives with potent anaerobic activity (e.g., moxifloxacin). Furthermore, the carbon atom at the C8 position can be replaced with nitrogen in naphthyridonic representatives with broad-spectrum activity (gemifloxacin, zabofloxacin) [244].

The third generation levofloxacin, and fourth generation moxifloxacin, are used against *Mycobacterium tuberculosis* [12,13]. The fourth generation exhibits broad-spectrum activity against Gram-positive and Gram-negative bacteria. Also, these representatives are active against anaerobes and atypical bacteria [243].

Jones et al. (2016) consider that avarofloxacin (acorafloxaxin, JNJ-Q2) is a new FQN from the fifth generation (chapter 8). This new compound is highly active against drug-resistant pathogens as MRSA, ciprofloxacin-resistant MRSA, and drug-resistant *Streptococcus pneumoniae* [248].

Although the mechanism of action of new FQNs(QNs) is based on the activity on the two target enzymes, DNA gyrase and topoisomerase IV, some particular aspects emerge from the structural and biological properties of the new compounds:

- the majority of the new representatives have a broad spectrum of activity, including activity against anaerobic bacteria (except nemonoxacin);
- the new representatives are active against many resistant bacteria (including resistant to FQNs); this is the main advantage of the newly approved compounds;
- some representatives are very active in the environment with acidic pH (delafloxacin, finafloxacin), this being an advantage over previous generations’ representatives;
- some representatives were approved only for a specific type of administration (topic); these are very effective in the treatment of targeted infections (besifloxacin, finafloxacin); for these compounds, there are numerous ongoing clinical trials for oral or parenteral administration;
- lascufloxacin has superior tissue penetration due to its high binding capacity to phosphatidylserine.

Given these aspects, we believe that there are premises to classify these new compounds in a new generation (the fifth). However, these new representatives still require supervision and further studies considering the fate of the many representatives withdrawn from previous generations due to the severe side-effects.

## 7. Antimicrobial Resistance to the Newer FQNs

Bacterial resistance to FQNs is a worldwide growing phenomenon; new resistant strains to FQNs have emerged in the last twenty years. The enhancement of bacterial resistance to FQNs will change patient management. This threatening phenomenon will produce changes in the therapeutic guidelines [249].

In this context, the newer FQNs aimed to reduce bacterial resistance in both humans and animals. However, the increase in bacterial resistance to FQNs has led to researchers’ efforts to understand resistance mechanisms and to identify new FQNs to combat the growing resistance. Mainly, the mechanisms of bacterial resistance to FQNs include: (1) mutations in topoisomerase II; (2) decreased drug absorption by upregulation of efflux pumps; and (3) plasmid-mediated resistance [62,63].

Mutations cause the most significant form of antimicrobial resistance in DNA gyrase and DNA topoisomerase IV. These mutations affect the interactions between FQNs and DNA enzymes [63]. Plasmid-mediated resistance encodes proteins that disrupt FQNs-enzyme interactions, increase FQNs efflux, or alter FQNs metabolism [250]. Chromosome-mediated resistance affects cellular efflux pumps, decreasing cellular concentrations of FQNs [251,252].

It is known that older FQNs act on a single target enzyme [253]. On the other hand, it is currently considered that newer FQNs drugs, such as besifloxacin [105], delafloxacin or zabofloxacin [254] can act on both DNA topoisomerases [255,256]. Thus, antimicrobial activity increases and the spontaneous occurrence of FQNs resistance is reduced [257]. For example, some studies on *Staphylococus pneumoniae* have concluded that besifloxacin has a higher inhibitory activity against DNA gyrase and DNA topoisomerase IV than ciprofloxacin and moxifloxacin. In the case of DNA gyrase, the inhibitory concentration of besifloxacin against *Staphylococus pneumoniae* was up to eight times lower comparing with moxifloxacin and 15 times lower comparing with ciprofloxacin [126]. These results suggest that besifloxacin is less affected by target enzymes mutations than earlier FQNs [258]. The same conclusion was presented by Roychoudhury et al., following in vitro study with nemonoxacin on resistant *Streptococcus pneumoniae* [259].

It was shown that drug efflux pumps do not contribute significantly to antibiotic resistance for newer FQNs, such as besifloxacin [260]. Besifloxacin is administered only ophthalmically. This can be considered an advantage due to the less likely risk of the development of microbial resistance [101].

Other in vitro studies have also shown that MRSA is less likely to develop resistance to delafloxacin compared to older FQNs. Regarding nadifloxacin, Alba et al. [182] demonstrated no increase in resistance of *Propionibacterium acnes*, *Staphylococcus aureus* (MRSA and MSSA) and *Staphylococcus epidermidis*, showing much better antimicrobial activity compared to other antibiotics. The reduction in resistance to nadifloxacin appears probably because it is not influenced by overexpression of the NorA efflux pump on the bacterial cell membrane [88].

Predicting resistance potential is based on some essential aspects. Among them are determinants of bacterial resistance, dual activity on target enzymes, and effects on bacterial efflux systems. In addition, the newer FQNs seem to have the advantage to maintain concentrations higher than MIC of first-step resistant mutants. The detection of all gyrA mutations which confer resistance is helpful in rapid molecular diagnosis of FQN resistance [261]. Mismatch amplification mutation assay-polymerase chain reaction (MAMA-PCR) technique may serve as a tool to identify the multiple point mutations in the FQN resistance in Gram-negative bacteria [262].

Therefore, the double targeting and low resistance of bacteria are specific features of the new FQNs. Future studies are needed to complete the description of the resistance mechanism of new FQNs.

## 8. Compounds in Development Based on Antibacterial QNs Structures

There are numerous compounds in development that have been included in several recently published review articles [247,263]. The discovery of new potential drugs is in continuous progress. Below are briefly presented some relevant compounds under development.

### 8.1. Avarofloxacin (Acorafloxacin)

Avarofloxacin (acorafloxacin, JNJ-Q2) or (7-[3-[2-Amino-1(*E*)-fluoroethylidene]piperidin-1-yl]-1-cyclopropyl-6-fluoro-8-methoxy-4-oxo-1,4-dihydroquinoline-3-carboxylic acid) [143,220] is a new FQN with a zwitterionic aminoethylidenylpiperidine structure [233]. It is currently undergoing clinical testing (phase III) to treat acute bacterial skin and skin-structure infections, CAP. It has shown improved antibacterial effectiveness against pathogens resistant to current FQNs [143].

It has antibacterial activity against a wide range of Gram-positive bacteria, including *Streptococcus pneumoniae*, MRSA, *Enterococcus* sp., *Escherichia coli*, *Klebsiella* spp., *Haemophilus influenzae* and *Pseudomonas aeruginosa* making it more potent than previously used FQNs [264].

Avarofloxacin can be administered orally and parenterally; the bioavailability is around 65% in parenteral oral administration. The fact that avarofloxacin is accessible in both parenteral and oral formulations sets it apart from several other MRSA treatments that are only available via injection [265].

In vitro investigations show that avarofloxacin has significant efficacy against pathogens including *Staphylococcus aureus* and *Streptococcus pneumoniae*, which cause acute bacterial skin and skin structure infections and community-acquired bacterial pneumonia; it was also demonstrated to have a more considerable resistance barrier than other drugs in the class, and it is still effective against drug-resistant organisms like MRSA, ciprofloxacin-resistant MRSA. Avarofloxacin was found to be as effective as linezolid for bacterial skin and skin structure infections and moxifloxacin for community-acquired bacterial pneumonia in two Phase II investigations [248]. Avarofloxacin has been granted Qualified Infectious Disease Product and Fast Track designations from the FDA [266].

### 8.2. Other Derivatives of Antibacterial QNs

Darehkordi et al. (2011) used N-substituted trifluoroacetimidoyl chlorides to synthesize piperazinyl QN derivatives. Out of the obtained compounds, two exhibited superior antibacterial activity against strains of *Escherichia coli*, *Klebsiella pneumonia* (compared to ciprofloxacin) and *Staphylococcus aureus* (compared to vancomycin) [267].

Sweelmeen et al. (2019) synthesized a novel derivative with antimicrobial potential (7-chloro-1-alkyl-6-fluoro-8-nitro-4-oxo-1,4-dihydroquinoline-3-carboxylic acid). This compound has been shown to be active against *Pseudomonas aeruginosa, Staphylococcus aureus, and Streptococcus agalactiae* [268]. In a review article, Zhang Bo (2014) highlighted different series of QN derivatives with antifungal potential in terms of structure-activity relationship: 2-quinolone, 4-quinolone, and FQN derivatives and FQN-metal complexes [263]. Lapointe et al. (2021) recently published the discovery and optimization of a novel series of compounds that inhibit the two bacterial target enzymes and stabilize the DNA cleavage complexes [269].

### 8.3. Hybrids

Numerous studies have aimed to obtain hybrid compounds that combine the properties of FQN with other types of active molecules [237,241,270,271]. In addition to broadening the spectrum of activity, the decrease in susceptibility to the installation of bacterial resistance is also pursued. Several hybrids were obtained with other antibiotics (e.g., oxazolidinones, tetracyclines, and aminoglycosides).

Gordeev et al. (2003) synthesized several compounds that incorporated pharmacophore structures of FQNs and oxazolidinones and demonstrated superior potency to linezolid against Gram-positive and Gram-negative bacteria, even for linezolid and ciprofloxacin-resistant strains of *Staphylococcus aureus* and *Enterococcus faecium*. The mechanism of action combined the inhibition of protein synthesis but also of DNA gyrase and topoisomerase IV [272]. Sriram et al. (2007) combined representatives from the tetracyclines class (tetracycline, oxytetracycline, and minocycline) with the secondary amino (piperazine) function of FQNs (norfloxacin, lomefloxacin, ciprofloxacin, and gatifloxacin). The results revealed anti-HIV and antitubercular activities, most significant for one of the compounds (minocycline-lomefloxacin derivative), making it a promising candidate in treating patients with HIV-1, co-infected with *Mycobacterium tuberculosis* [273].

Pokrovskaya et al. (2009) synthesized a series of hybrids with ciprofloxacin and neomycin. The antibacterial activity of most of the synthesized compounds was significantly higher on *Escherichia coli* and *Bacillus subtilis*, compared to that of the two free antibiotics. This case also showed that the combinations presented a dual mechanism of action, namely the inhibition of protein synthesis and target enzymes of FQNs [274]. Gorityala et al. (2016) studied an antibacterial hybrid consisting of moxifloxacin and tobramycin that acts against multidrug-resistant strains of *Pseudomonas aeruginosa,* by improving membrane permeability and reducing efflux [275]. Shavit et al. (2017) synthesized a series of hybrids composed of ciprofloxacin and kanamycin A, which showed superior action on Gram-negative bacteria. These hybrids delayed the emergence of resistance for strains of *Escherichia coli* and *Bacillus subtilis* compared to the 1:1 mixture of the two antibiotics [276].

In addition to the hybridization of antibacterial QNs with other antibiotics, several studies have included different types of drugs with biological potential in the design of hybrids. For example, Chugunova et al. (2016) synthesized a series of FQN hybrids with benzofuroxane derivatives. Some combinations showed superior antibacterial activity on *Bacillus cereus* 8035 strains compared to the free FQN [270]. Wang YN et al. (2018) synthesized a series of hybrids between QN derivatives and benzimidazole. One of the compounds showed unusual activity on the resistant strains of *Pseudomonas aeruginosa* and *Candida tropicalis* strains. It also caused a decrease in the resistance of *Pseudomonas aeruginosa*, compared to norfloxacin [277].

A series of 34 clinafloxacin-azole conjugates were synthesized and tested in vitro against *Mycobacterium tuberculosis* (H37Rv) and other Gram-negative and Gram-positive bacteria. A particular conjugate (TM2l) has been the most promising delimited in terms of a great activity against *Mycobacterium tuberculosis* (MIC = 0.29 μM), good safety predicted profile, and good drug-likeness values [124].

Yi-Lei Fan et al. (2018) review the numerous FQN derivatives as antituberculosis agents. Among them are FQN-isatin hybrids, FQN-azole hybrids, FQN-amide/azetidinone derivatives, FQN-quinoline/phenanthridine hybrids, FQN-hydrazone/hydrazide hybrids, dimeric FQN derivatives, FQN-oxime hybrids, FQN-sugar/coumarin/dihydroartemisinin/tetracycline hybrids, and other FQN derivatives [241].

A whole decade has been reviewed from the perspective of hybrid compounds and dual-action molecules by Fedorowicz and Sączewski (2018) [271].

## 9. Concerning Side Effects

Currently, FQNs are a valuable class of drugs used to treat infections with Gram-positive and Gram-negative bacteria (Table S1—Supplementary Material). However, the new generations of FQNs have a broad spectrum of activity, including drug-resistant bacterial species (see recent authorized FQNs previously discussed). Unfortunately, this antibacterial class has been overused in therapy over time. It is known that FQNs could produce a series of severe side effects, which vary from one representative to another, mainly if they are not used judiciously [18,278,279,280,281,282,283]. These side effects occur at the gastrointestinal tract level (nausea and diarrhea), central nervous system (headache, dizziness, confusion, seizures, and insomnia), joints (Achilles tendon rupture), and muscles (neuromuscular blocking activity), cardiovascular system (QT prolongation and arrhythmias). Also, the FQNs could produce dysglycemia, hepatotoxicity, renal toxicity, phototoxicity, rush, anaphylactoid reactions, and anaphylaxis [11,18,282,284,285,286,287].

FDA has approved labeling changes of FQNs (black box warning) [288,289] and has issued a series of warnings about FQNs side effects [290], as tendinopathy and tendon rupture [291], aortic rupture or tears [292], and the negative impact on mental health and glucose homeostasis (dysglycemia) [293]. EMA has also issued similar warnings, suspensions, or restrictions of FQNs due to their potentially permanent side effects [294,295,296,297,298].

However, FQNs proved to be a beneficial antibacterial class, safe in the low doses and short course [281]; these drugs have potential side effects, especially in long or high doses, limiting their use. Therefore, FQNs of the new generations must be used responsibly, only in severe life-threatening infections with no alternative treatment options [281,288,299,300].

## 10. Conclusions

Antibacterial QNs had developed spectacularly over time, many compounds being approved and used successfully in therapy. Therefore, identifying novel antibacterial compounds has been a priority in recent years to produce effective treatments against bacteria that have gained resistance to classic FQNs. However, more information on efficacy against multidrug-resistant organisms is still needed, as these new medications are primarily aimed at these resistant strains.

Structure-activity relationship investigations were crucial in identifying substituents with a high affinity for binding to two target enzymes, the DNA gyrase and the topoisomerase IV enzymes. We have critically analyzed the structural changes in the new compounds compared to analogues from previous generations. Substitutes and combinations of substituents on the QN nucleus proved to confer to these new FQNs an acceptable safety profile by exceeding the possible side-effects identified in older compounds. These new representatives were highlighted by a broad spectrum of activity, including activity against anaerobic bacteria (except nemonoxacin). Many resistant bacteria (including resistant to FQNs) are susceptible to these compounds. Delafloxacin and finafloxacin have the advantage of being very active in an environment with acidic pH. Lascufloxacin has superior tissue penetration due to its high binding capacity to phosphatidylserine. Besifloxacin and finafloxacin were approved only for topic administration and are very effective in treating targeted infections. Thus, several positive aspects are added to the fourth generation FQNs, characteristics that can be the basis of a new generation (the fifth).

New molecules are in different phases of research, derivatives of FQNs (e.g., levonadifloxacin, avarofloxacin), and their conjugates or hybrids. This class of antimicrobials remains in the attention of researchers focused on developing new drugs efficient against resistant pathogens. However, the maximum therapeutic potential of this antimicrobials class has not been reached yet.

## Figures and Tables

**Table 1:** The representatives of the class of antibacterial QNs and their approval in therapy.

^1^ Year	Generation	Antibacterial QNs	Producer	Use and Status	References
1962	1st	Nalidixic acid	Lappin/Sterling Drug	Human and veterinary	[14,15]
1966	1st	Oxolinic acid	Warner-Lambert	Withdrawn	[14,15]
1967	1st	Piromidic acid	Dainippon	Withdrawn	[14,15]
1970	1st	Cinoxacin	Eli Lilly	Withdrawn	[14]
1973	1st	Flumequine	Riker	Veterinary	[14,15]
1974	1st	Pipemidic acid	Dainippon	Withdrawn	[14]
1978	2nd	Norfloxacin	Kyorin	Human and veterinary Approved by the FDA in 1986	[14]
1979	2nd	Pefloxacin	Roger Bellan (Rhône Poulenc)	Approved in France since 1985; approved in some EU countries	[14,15,16,17]
1980	2nd	Enoxacin	Dainippon	Withdrawn	[3,14,15]
1981	2nd	Fleroxacin	Kyorin	Introduced in therapy in 1987, withdrawn in 1990	[14,15,18]
1982	2nd	Ofloxacin ^2^	Daiichi	Human and veterinary, Approved by the FDA in 1990	[14,15]
1984	2nd	Temafloxacin	Abbott Laboratories	Approved by the FDA and also withdrawn in 1992	[8,18,19]
1985	2nd	Lomefloxacin ^2^	Hokuriku Pharm.	Approved by the FDA in 1992, then withdrawn	[3]
1985	2nd	Tosufloxacin	Taisho-Toyama Chemistry, Abbott	Veterinary (Japan)	[8,15,20]
1987	2nd	Ciprofloxacin	Bayer AG	Human and veterinary, Approved by the FDA in 1987	[14,15]
1987	3rd	Enrofloxacin	Bayer AG	Veterinary	[14,15,21]
1987	3rd	Sparfloxacin ^3^	Dainippon	Approved by the FDA in 1996, then withdrawn	[3,14,15]
1987	4th	Prulifloxacin	Nippon	Approved only in Japan	[14,22,23]
1987	3rd	Orbifloxacin ^3^	Dainippon/Schering	Veterinary	[14,24]
1989	2nd	Nadifloxacin ^2^	Otsuka Pharmaceuticals	Approved in Japan in 1993 and 1998, approved by the EMA in 2000, topical use	[25,26,27]
1989	3rd	Grepafloxacin ^2^	Warner Lambert/Glaxo Wellcome	Withdrawn in 1999	[3,15]
1990	3rd	Clinafloxacin	Parke-Davis Pharmaceutical	Withdrawn in 1999	[3,14]
1991	3rd	Danofloxacin	Pfizer	Veterinary	[14,15,28]
1992	4th	Trovafloxacin	Pfizer	Withdrawn in 2001	[3,14,15]
1994	3rd	Levofloxacin ^4^	Daiichi	Approved by the FDA in 1996	[14,15,29]
1994	2nd	Sarafloxacin	Abbott Laboratories/Fort Dodge	Veterinary, withdrawn in 2001	[14,15,30,31]
1995	3rd	Balofloxacin	Choongwae Pharma	Approved by the Korean FDA in 2001	[23,32]
1995	3rd	Marbofloxacin	Vetoquinol/Pfizer	Veterinary	[14,15,33]
1995	4th	Moxifloxacin ^4^	Bayer AG	Approved by the FDA in 1999	[3,29,34]
1996	2nd	Difloxacin	Fort Dodge	Veterinary	[14]
1998	3rd	Pradofloxacin	Bayer Animal Health GmbH	Approved by the EMA in 2011, and FDA in 2013; veterinary use	[35,36]
1999	4th	Delafloxacin	Abbott Laboratories, Melinta Therapeutics (former Rib-X Pharmaceuticals)	Approved by the FDA in 2017	[37,38,39,40,41,42]
1999	3rd	Gatifloxacin ^2^	Kyorin/Bristol-Myers Squibb	Approved by the FDA in 1999, withdrawn in 2006	[3,25,43,44]
1999	4th	Gemifloxacin	Smith-Kline Beecham	Approved by the FDA in 2003 Withdrawn in 2009 (by EMA)	[3,25,29,45]
2000	4th	Besifloxacin ^4^	SSP Co. Ltd., Japonia	Approved by the FDA in 2009	[46]
2000	4th	Finafloxacin ^3^	Bayer HealthCare Pharmaceuticals, Byk Gulden, MerLion Pharmaceuticals	Approved by the FDA in 2017	[42,47,48,49,50]
2003	4th	Garenoxacin	Toyama Chemical Co., Ltd./Schering Plough	Approved by the FDA and EMA in 2006; withdrawn in 2007	[3,29,51,52]
2004	4th	Nemonoxacin	TaiGen Biotechnology	Approved in Taiwan in 2014	[53,54]
-	4th	Zabofloxacin	Dong Wha Pharm. Co. Ltd.	Approved in Soth Korea in 2015	[55,56]
-	4th	Sitafloxacin	Daiichi Sankyo Co., Japan	Approved in Japan in 2008, in Thailand in 2012	[57,58,59]

^1^ first year reported, ^2^ racemic, ^3^ diastereoisomers, ^4^ *R* or *S* isomer.

**Table 2:** Classification into generations of the main FQNs for human use in therapy based on antibacterial spectrum and therapeutic indications (FDA and EMA approved).

Generation	Compounds	Antibacterial Spectrum	Therapeutic Indications/Pharmacokinetics, Administration	Ref.
1st	Nalidixic acid	Gram-negative pathogens—Enterobacteriaceae (without Pseudomonas species)	Uncomplicated urinary tract infections; Oral administration, low serum and tissue concentrations, renal elimination, short half-life.	[3,8,82,83]
2nd	Norfloxacin Ciprofloxacin Ofloxacin Pefloxacin	Enterobacteriaceae; Some atypical pathogens; Pseudomonas aeruginosa (ciprofloxacin only); Some Gram-positive pathogens (including Streptococcus pneumoniae), moderate activity on Staphylococcus aureus.	Uncomplicated and complicated urinary tract infections, pyelonephritis, sexually transmitted diseases, prostatitis, skin and tissue infections; Oral administration, low serum and tissue concentrations (only for norfloxacin); Oral and parenteral administration, high concentrations in serum and tissues, longer half-life.	[3,5,83,84,85,86]
2nd	Nadifloxacin (topical use)	Gram-positive (including methicillin-resistant Staphylococcus aureus (MRSA) and coagulase-negative staphylococci), aerobic Gram-negative, and anaerobic pathogens.	Treatment of acne vulgaris and other skin infections. Topical use, 1% cream.	[26,87,88,89]
3rd	Levofloxacin	Enterobacteriaceae; Atypical pathogens; Penicillin-resistant Streptococcus pneumoniae.	Acute and chronic bronchitis, exacerbated forms, acquired pneumonia (nosocomial); Oral and parenteral administration, high serum and tissue concentrations, long half-life (6–8 h). Ophthalmic use (0.5% ophthalmic solution).	[29,86,90,91]
3rd	Gatifloxacin (ophthalmic use)	Broad-spectrum including Staphylococcus aureus, Streptococcus species, and Gram-negative pathogens	Bacterial conjunctivitis, ophthalmic use (0.3% or 0.5% ophthalmic solution).	[43]
4th	Moxifloxacin	Enterobacteriaceae; Atypical pathogens; Pseudomonas aeruginosa; Streptococci; MRSA; Anaerobic pathogens. Others: Chlamydophila pneumoniae, Mycoplasma pneumonia	Sexually transmitted diseases, prostatitis, skin and tissue infections, acute and chronic bronchitis, exacerbated forms, acquired pneumonia (nosocomial), intra-abdominal infections, gynecological infections; bacterial conjunctivitis. Oral, parenteral, and ophthalmic administration (0.5%), high serum and tissue concentrations, long half-life (8–16 h).	[34,92,93]
4th	Delafloxacin	Gram-positive (including methicillin-resistant Staphylococcus aureus) and Gram-negative pathogens	Bacterial skin and skin structure infections. Oral and intravenous administration, oral bioavailability 58.8%, plasma protein binding 84%, mean half-life 4.2–8.5 h (oral), and 3.7 h (intravenous).	[39,41,94]
4th	Besifloxacin (topical, ophtalmic administration)	Streptococcus pneumonia, Staphylococcus epidermidis, Staphylococcus aureus, Haemophilus influenza, Moraxella catarrhalis, Corynebacterium spp	Bacterial conjunctivitis. Ophthalmic suspension (0.6%).	[46,95,96,97]
4th	Finafloxacin (topical, otic administration)	Broad-spectrum activity (very active against Pseudomonas aeruginosa, and Staphylococcus aureus)	Acute otitis externa. Otic suspension (0.3%)	[98,99,100]

**Table 10:** Essential moieties on the QN nucleus for the newer compounds.

Position (Atom)	Substituent	Moieties’ Chemical Structure	Antibacterial QN
1 (C)	Cyclopropyl	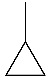	Besifloxacin Finafloxacin Nemonoxacin Zabofloxacin
	6-Amino-3,5-difluoropyridinyl	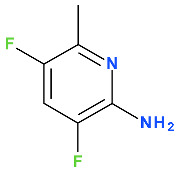	Delafloxacin
6 (C)	Fluor	–F	All compounds, except nemonoxacin
7 (C)	3-Aminoazepane	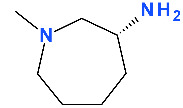	Besifloxacin
	3-Hydroxyazetidinyl	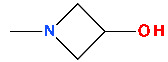	Delafloxacin
	Pyrrolo-oxazinyl	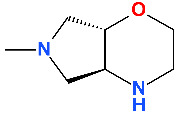	Finafloxacin
	3-Amino-5-methylpiperidinyl	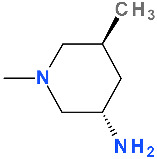	Nemonoxacin
	2,6-Diazaspiro[3.4]octanyl	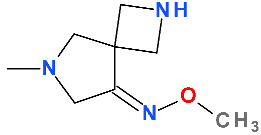	Zabofloxacin
	[(Cyclopropylamino)methyl]-4-fluoropyrrolidinyl	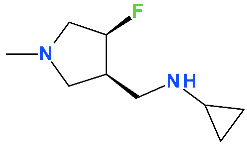	Lascufloxacin
8 (7 ^1^) (C)	4-Hydroxypiperidinyl	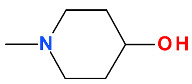	Nadifloxacin ^2^ and levonadifloxacin
8 (C)	Chloride	–Cl	Besifloxacin Delafloxacin
	Cyano group	–CN	Finafloxacin
	Methoxy	–O–CH_3_	Nemonoxacin
8 (N)	Lack of substituent	none	Zabofloxacin
9 (8 ^3^)	Condensation of quinolone nucleus with methylpiperidine	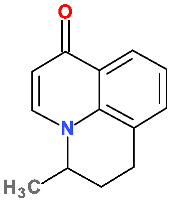	Nadifloxacin

^1^ Similar to C7 on QN nucleus; ^2^ Tricyclic structure; ^3^ Similar to C8 on QN nucleus.

## Supplementary Materials

The following are available online at https://www.mdpi.com/article/10.3390/pharmaceutics13081289/s1, Figure S1: Chemical structures of other FQNs from different generations, Table S1: Activity spectrum of major QNs approved for use in therapy after 2000.
