# Supplementary material for: Structural Characterization of the Millennial Antibacterial (Fluoro)Quinolones—Shaping the Fifth Generation
Source: Pharmaceutics. 2021 Aug 18;13(8):1289. doi: 10.3390/pharmaceutics13081289 (PMC8399897; doi:10.3390/pharmaceutics13081289)
Supplement: Supplementary file 1 [file pharmaceutics-13-01289-s001.zip › pharmaceutics-1313721-supplementary.pdf]

*pharmaceutics*

MDPI

# Supplementary Materials: Structural characterization of the millennial antibacterial (fluoro)quinolones - shaping the fifth generation

**Aura Rusu, Ioana-Andreea Lungu, Octavia-Laura Moldovan, Corneliu Tanase and Gabriel Hancu**

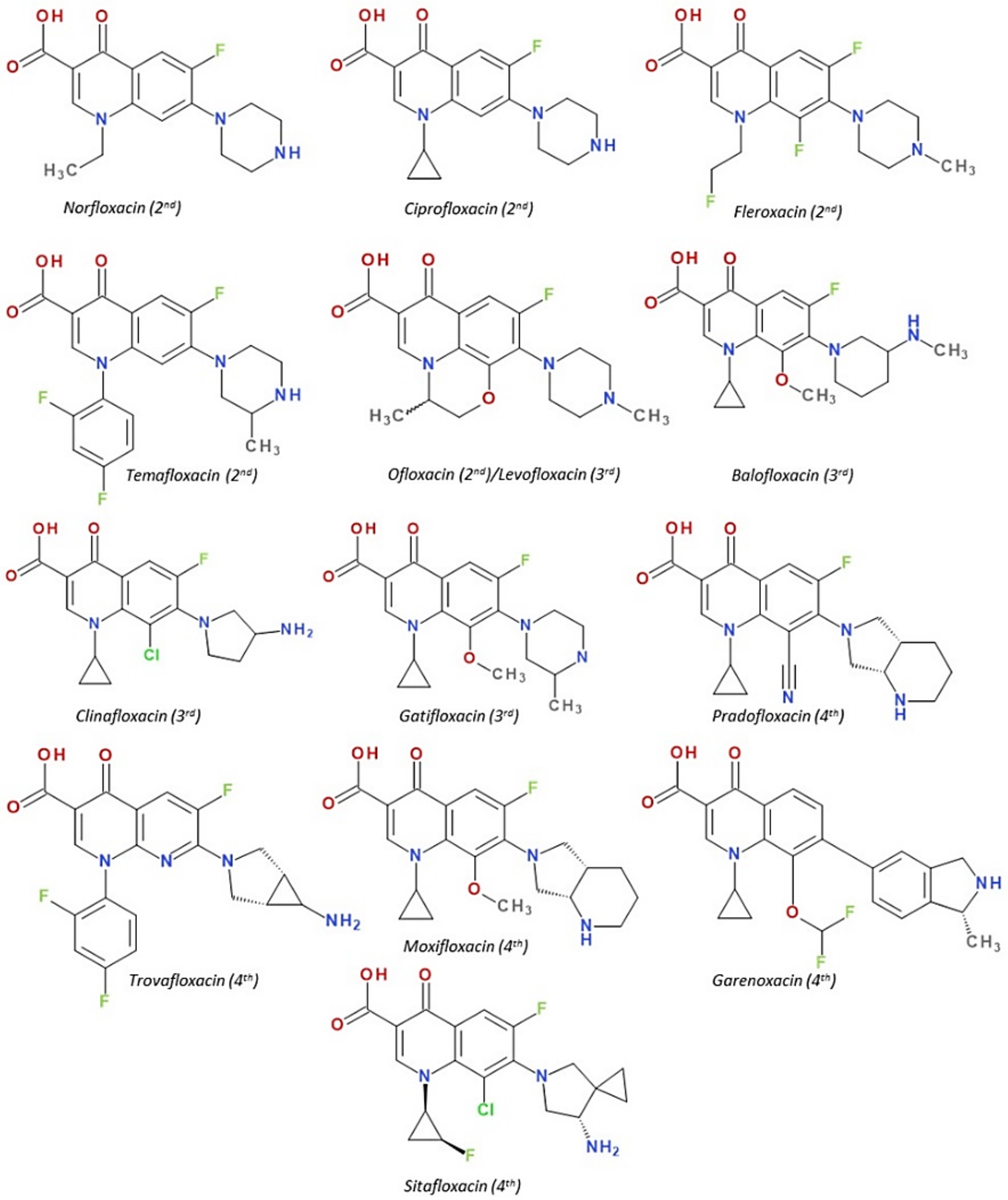


**Figure S1.** Chemical structures of other FQNs from different generations.

**Table S1.** Activity spectrum of major QNs approved for use in therapy after 2000.

| QNs | Gram-positive pathogens | Gram-negative pathogens | Other pathogens | References |
|---|---|---|---|---|
| Besifloxacin | ***Aerobes***<br>**-Cocci:** *Staphylococcus aureus and MRSA; Staphylococcus epidermidis (MRSE); Staphylococcus hominis; Staphylococcus lugdunensis; Staphylococcus warneri; Staphylococcus saprophyticus; Staphylococcus haemolyticus; Streptococcus mitis group; Streptococcus oralis; Streptococcus pneumoniae; Streptococcus salivarius; Streptococcus agalactiae; Streptococcus pyogenes; Streptococcus viridans*<br>***Facultative anaerobes***<br>**-Cocci:** *Aerococcus viridans*<br>**-Bacilli:** *Propionibacterium acnes*<br>***Anaerobes***<br>**-Cocci:** *Enterococcus faecalis; Enterococcus faecium; Peptostreptococcus spp;*<br>-**Bacilli:** *CDC coryneform group G; Corynebacterium pseudodiphtheriticum; Corynebacterium striatum; Listeria monocytogens; Clostridium perfringens* | ***Aerobes***<br>**-Cocci:** *Neisseria meningitidis;*<br>**-Bacilli:** *Legionella pneumophila; Stenotrophomonas maltophilia; Moraxella lacunata; Moraxella catarrhalis; Acinetobacter baumanii; Acinetobacter calcoaceticus; Pseudomonas aeruginosa; Brevundimonas diminuta*<br>***Facultative anaerobes***<br>**-Bacilli:** *Haemophilus influenzae; Serratia marcesnes; Citrobacter freundii; Citrobacter koseri; Klebsiela pneumonie; Klebsiella oxytoca; Enterobacter aerogenes; Enterobacter cloacae; Morganella morganii; Proteus miriabilis; Proteus vulgaris; Escherichia coli;*<br>***Anaerobes***<br>-**Bacilli:** *Bacteroides fragilis; Fusobacterium spp; Prevotella spp;* | **-Bacilli:** *Mycobacterium chelonae; Mycobacterium fortuitum* | [1–13] |
| Delafloxacin | ***Aerobes***<br>**-Cocci:** *Staphylococcus aureus* (including MRSA and methicillin-susceptible isolates); *Staphylococcus haemolyticus; Staphylococcus hominis; Staphylococcus lugdunensis; Staphylococcus epidermidis; Streptococcus agalactiae; Streptococcus anginosus spp. (Streptococcus anginosus;*<br>*Streptococcus intermedius; Streptococcus constellatus); Streptococcus dysgalactiae; Streptococcus pyogenes; Streptococcus pneumoniae* (including isolates resistant to penicillin and macrolides); *Streptococcus mitis group* (including *Streptococcus cristatus; Streptococcus gordonii;* | ***Aerobes***<br>**-Cocci:** *Neisseria gonorrhoeae; Neisseria meningitidis;*<br>**-Bacilli:** *Pseudomonas aeruginosa;*<br>*Moraxella catarrhalis; Legionella pneumophila; Acinetobacter spp; Stenotrophomonas maltophilia*<br>***Facultative anaerobes***<br>**-Bacilli:** *Escherichia coli, Escherichia aerogenes; Escherichia faecalis; Enterobacter cloacae; Salmonella typhimurium; Salmonella aureus; Salmonella epidermidis; Salmonella pneumonia; Salmonella haemolyticus; Klebsiella pneumoniae; Klebsiella oxytoca ; Proteus mirabilis ; Haemophilus influenzae (including β-lactam-resistant isolates); Yersinia pestis*<br>***Anaerobes*** | **-Bacilli:** *Mycobacterium tuberculosis*<br>***-Aerobe-microaero-philes:*** *Helicobacter pylori*<br>***-Intracelullar bacteria***<br>*Chlamydia pneumoniae;* Mycoplasmas *(Myco-plasma pneumoniae)* and ureaplasmas | [14–27] |

| QNs | Gram-positive pathogens | Gram-negative pathogens | Other pathogens | References |
|---|---|---|---|---|
| | *Streptococcus oralis; Streptococcus mitis; Streptococcus sanguinis)*<br>***Facultative anaerobes***<br>**-Cocci:** -<br>**-Bacilli:** *Bacillus subtilis; Bacillus cereus; Propionibacterium spp;*<br>***Anaerobes***<br>**-Cocci:** *Enterococcus faecalis;*<br>*Peptostreptococcus spp;*<br>**-Bacilli:** *Clostridium Difficile; Clostridium perfringens* | **-Cocci:** *Veillonella sp.*<br>**-Bacilli:** *Bacterioides Fragilis; Fusobacterium spp; Prevotella spp; Porphyromonas sp;* | | |
| Finafloxacin | ***Aerobes***<br>**-Cocci:** *Staphylococcus aureus* (MRSA, MSSA); *Staphylococcus epidermidis; Streptococcus pneumoniae; Beta-hemolytic streptococci;*<br>**-Bacilli:** *Bacillus anthracis;*<br>***Facultative anaerobes***<br>**-Cocci:** -<br>**-Bacilli:** *Propionibacterium Acnes;*<br>***Anaerobes***<br>**-Cocci:** *Enterococcus faecalis; Peptostreptococcus anaerobius*<br>**-Bacilli:** *Corynebacterium Amycolatum; Clostridium perfringens; Listeria monocitogens* | ***Aerobes***<br>**-Cocci:** *Legionella pneumophila; Pseudomonas aeruginosa (Ciprofloxacin-resistant and Ciprofloxacin-susceptible Pseudomonas. Aeruginosa); Pseudomonas otitidis; Burkholderia pseudomallei; Burkholderia mallei; Acinetobacter baumannii; Moraxella Catarrhalis; Stenotrophomonas maltophilia;*<br>**-Coccobacilli:** *Francisella tularensis*<br>**-Bacilli:** -<br>***Facultative anaerobes***<br>**-Bacilli:** *Escherichia coli; Klebsiella pneumonia; Proteus vulgaris; Proteus mirabilis; Haemophillus influenzae; Enterobacter cloacae; Proteus mirabilis; Providencia spp, Salmonella spp, Serratia marcescens; Yersinia pestis*<br>***Anaerobes***<br>**-Cocci:** -<br>**-Bacilli:** *Bacterioides fragilis* | **-Bacilli:** *Mycobacterium tuberculosis*<br>***-Aerobe-microaero-philes:*** *Helicobacter pylori*<br>***-Intracelullar bacteria***<br>*Chlamydia pneumoniae; Mycoplasma pneumoniae* | [17,28–38] |
| Lascufloxacin | ***Aerobes***<br>**-Cocci:** *Staphylococcus aureus(MRSA, MSSA),* | ***Aerobes***<br>**-Cocci:** -;<br>**-Bacilli:** *Moraxella catarrhalis; Legionella pneumophila; Acinetobacter spp* | ***-Aerobe-microaerophiles:*** | [39–43] |

| QNs | Gram-positive pathogens | Gram-negative pathogens | Other pathogens | References |
|---|---|---|---|---|
| | *Staphylococcus epidermidis; Staphylococcus saccharolyticus; Streptococcus pyogenes; Streptococcus constellatus ; Streptococcus intermedius; Streptococcus agalactiae; Streptococcus anginosus Streptococcus pneumoniae* (penicillin-susceptible and penicillin-resistant *S. pneumoniae)*<br>**-Bacilli:** -<br>***Facultative anaerobes***<br>**-Cocci:** -<br>**-Bacilli:** -<br>***Anaerobes***<br>**-Cocci:** *Enterococcus faecalis; Peptostreptococcus anaerobius; Finegoldia magna; Peptoniphilus asaccharolyticus*<br>**-Coccobacilli:** *Slackia exigua*<br>**-Bacilli:** *Clostridium clostridioforme; Clostridioides difficile; Clostridium perfringens; Bulleidia extructa; Atopobium parvulum; Actinomyces odontolyticus* | ***Facultative anaerobes***<br>**-Bacilli:** *Klebsiella spp; Escherichia coli; Haemophilus influenzae; Enterobacter spp; Capnocytophaga ochracea;*<br>***Anaerobes***<br>**-Cocci:** *Veillonella spp*<br>**-Bacilli:** *Prevotella spp (Prevotella baroniae; Prevotella buccae; Prevotella corporis; Prevotella denticola; Prevotella intermedia; Prevotella melaninogenica; Prevotella oralis; Prevotella oris); Bacteroides fragilis; Bacteroides thetaiotaomicron; Fusobacterium necrophorum; Fusobacterium nucleatum; Porphyromonas asaccharolytica; Porphyromonas gingivalis* | *Campylobacter gracilis; Campylobacter ureolyticus*<br>***-Intracelullar bacteria***<br>*Mycoplasma pneumoniae* | |
| Nadifloxacin/ *Levonadifloxacin | ***Aerobes***<br>**-Cocci:** *Staphylococcus aureus* (MRSA)*; Coagulase-negative staphylococci: Staphylococcus epidermidis; Streptococcus spp;* *Quinolone-resistant *Staphylococcus aureus;* *Macrolide- and penicillin-resistant *Streptococcus pneumoniae; *Streptococcus pyogenes*<br>**-Bacilli:** -<br>***Facultative anaerobes***<br>**-Cocci:** -<br>**-Bacilli:** *Propionibacterium spp (P. acnes; P. granulosum)*<br>***Anaerobes***<br>**-Cocci:** -<br>-**Bacilli:** - | ***Aerobes***<br>**-Cocci:** -<br>**-Bacilli:** **Moraxella catarrhalis*<br>***Facultative anaerobes***<br>**-Bacilli:** **Haemophilus influenzae*<br>***Anaerobes***<br>**-Cocci:** -<br>-**Bacilli: -** | - | [44–48] |
| Nemonoxacin | ***Aerobes*** | ***Aerobes***<br>**-Cocci:** *Neisseria gonorrhoeae;* | **-Bacilli:** *Mycobacterium tuberculosis* | [17,49–57] |

| QNs | Gram-positive pathogens | Gram-negative pathogens | Other pathogens | References |
| --- | --- | --- | --- | --- |
| | **-Cocci:** *Staphylococcus aureus* (MSSA and MRSA - ciprofloxacin-susceptible and ciprofloxacin-resistant); *Staphylococcus epidermidis* (Methicillin susceptible *Staphylococcus epidermidis* (MSSE) and methicillin-resistant *S. epidermidis* (MRSE)); *Staphylococcus Capitis; Streptococcus pneumoniae* (Penicillin resistant and fluoroquinolone-resistant *Streptococcus pneumoniae); Streptococcus pyogenes; Streptococcus agalactiae; Viridans group streptococci*<br>**-Bacilli:** *Nocardia spp. (Nocardia brasiliensis ; Nocardia cyriacigeorgica ; Nocardia asteroides ; Nocardia farcinica)*<br>***Facultative anaerobes***<br>**-Cocci:** -<br>**-Bacilli:** -<br>***Anaerobes***<br>**-Cocci:** *Enterococcus faecium; Enterococcus faecalis*<br>-**Bacilli:** *Clostridium difficile* | **-Bacilli:** *Pseudomonas Aeruginosa; Legionella pneumophila; Moraxella catarrhalis; Stenotrophomonas maltophilia; Acinetobacter Baumannii*<br>***Facultative anaerobes***<br>**-Bacilli:** *Klebsiella pneumoniae; Klebsiella oxytoca; Haemophillus influenzae; Haemophillus Parainfluenzae; Enterobacter cloacae; Proteus mirabilis; Citrobacter freundii; Escherichia coli (ciprofloxacin-resistant E. coli, ciprofloxacin-susceptible E. coli)*<br>***Anaerobes***<br>**-Cocci:** -<br>**-Bacilli:** - | *(Mycobacterium tuberculosis,* multidrug-resistant and *M. tuberculosis* non-multidrug-resistant)<br>***-Aerobe-microaerophiles:*** *Helicobacter pylori*<br>***-Intracelullar bacteria***<br>*Chlamydia pneumoniae; Chlamydia trachomatis; Mycoplasma pneumoniae* | |
| Zabofloxacin | ***Aerobes***<br>**-Cocci:** *Staphylococcus aureus* MRSA; Methicillin-resistant coagulase-negative staphylococci; *Streptococcus pneumoniae* (Penicillin-sensitive *S. pneumoniae* and penicillin-resistant *S. pneumoniae); Streptococcus pyogenes*<br>**-Bacilli:** -<br>***Facultative anaerobes***<br>**-Cocci:** -<br>**-Bacilli:** -<br>***Anaerobes***<br>**-Cocci:** *Enterococcus Faecalis*<br>**-Bacilli:** - | ***Aerobes***<br>**-Cocci:** *Neisseria gonorrhoeae*<br>**-Bacilli:** *Pseudomonas aeruginosa; Moraxella catarrhalis*<br>***Facultative anaerobes***<br>**-Bacilli:** *Haemophilus influenzae; Klebsiella pneumoniae; Escherichia coli*<br>***Anaerobes***<br>**-Cocci:** -<br>-**Bacilli:** - | - | [17,58–62] |

## References

1. Mah, F.S.; Sanfilippo, C.M. Besifloxacin: Efficacy and Safety in Treatment and Prevention of Ocular Bacterial Infections. *Ophthalmol Ther* **2016**, *5*, 1–20, doi:10.1007/s40123-016-0046-6.
2. Haas, W.; Sanfilippo, C.M.; Hesje, C.K.; Morris, T.W. Contribution of the R8 Substituent to the in Vitro Antibacterial Potency of Besifloxacin and Comparator Ophthalmic Fluoroquinolones. *Clinical ophthalmology (Auckland, N.Z.)* **2013**, *7*, 821–30, doi:10.2147/OPTH.S44085.
3. Cambau, E.; Matrat, S.; Pan, X.-S.; Roth Dit Bettoni, R.; Corbel, C.; Aubry, A.; Lascols, C.; Driot, J.-Y.; Fisher, L.M. Target Specificity of the New Fluoroquinolone Besifloxacin in Streptococcus Pneumoniae, Staphylococcus Aureus and Escherichia Coli. *J Antimicrob Chemother* **2009**, *63*, 443–450, doi:10.1093/jac/dkn528.
4. Khimdas, S.; Visscher, K.L.; Hutnik, C.M.L. Besifloxacin Ophthalmic Suspension: Emerging Evidence of Its Therapeutic Value in Bacterial Conjunctivitis. *Ophthalmol Eye Dis* **2011**, *3*, 7–12, doi:10.4137/OED.S4102.
5. Sanfilippo, C.M.; Hesje, C.K.; Haas, W.; Morris, T.W. Topoisomerase Mutations That Are Associated with High-Level Resistance to Earlier Fluoroquinolones in Staphylococcus Aureus Have Less Effect on the Antibacterial Activity of Besifloxacin. *Chemotherapy* **2011**, *57*, 363–371, doi:10.1159/000330858.
6. Singh, C.L.; Singh, A.; Kumar, S.; Majumdar, D.K. BESIFLOXACIN THE FOURTH GENERATION FLUOROQUINOLONE: A REVIEW. *Journal of Drug Delivery and Therapeutics* **2014**, *4*, 39–44, doi:10.22270/jddt.v4i6.1012.
7. Totoli, E.G.; Nunes Salgado, H.R. Besifloxacin: A Critical Review of Its Characteristics, Properties, and Analytical Methods. *Crit. Rev. Anal. Chem.* **2018**, *48*, 132–142, doi:10.1080/10408347.2018.1429885.
8. DeCory, H.H.; Sanfilippo, C.M.; Proskin, H.M.; Blondeau, J.M. Characterization of Baseline Polybacterial versus Monobacterial Infections in Three Randomized Controlled Bacterial Conjunctivitis Trials and Microbial Outcomes with Besifloxacin Ophthalmic Suspension 0.6%. *PLoS One* **2020**, *15*, e0237603, doi:10.1371/journal.pone.0237603.
9. dos Santos, G.A.; Ferreira-Nunes, R.; Dalmolin, L.F.; dos Santos Re, A.C.; Vieira Anjos, J.L.; Mendanha, S.A.; Aires, C.P.; Lopez, R.F.V.; Cunha-Filho, M.; Gelfuso, G.M.; et al. Besifloxacin Liposomes with Positively Charged Additives for an Improved Topical Ocular Delivery. *Sci Rep* **2020**, *10*, 19285, doi:10.1038/s41598-020-76381-y.
10. Polat, H.K.; Pehlivan, S.B.; Ozkul, C.; Calamak, S.; Ozturk, N.; Aytekin, E.; Firat, A.; Ulubayram, K.; Kocabeyoglu, S.; Irkec, M.; et al. Development of Besifloxacin HCl Loaded Nanofibrous Ocular Inserts for the Treatment of Bacterial Keratitis: In Vitro, Ex Vivo and in Vivo Evaluation. *Int. J. Pharm.* **2020**, *585*, 119552, doi:10.1016/j.ijpharm.2020.119552.
11. Kassaee, S.N.; Mahboobian, M.M. Besifloxacin-Loaded Ocular Nanoemulsions: Design, Formulation and Efficacy Evaluation. *Drug Deliv. Transl. Res.*, doi:10.1007/s13346-021-00902-z.
12. FDA, N. 22308/S-013 Besifloxacin Label.
13. Besifloxacin Available online: https://go.drugbank.com/drugs/DB06771 (accessed on 1 June 2021).
14. Hanselmann, R.; Johnson, G.; Reeve, M.M.; Huang, S.-T. Identification and Suppression of a Dimer Impurity in the Development of Delafloxacin. *Org. Process Res. Dev.* **2009**, *13*, 54–59, doi:10.1021/op800238q.
15. Lemaire, S.; Tulkens, P.M.; Van Bambeke, F. Contrasting Effects of Acidic PH on the Extracellular and Intracellular Activities of the Anti-Gram-Positive Fluoroquinolones Moxifloxacin and Delafloxacin against Staphylococcus Aureus. *Antimicrob Agents Chemother* **2011**, *55*, 649–658, doi:10.1128/AAC.01201-10.
16. Van Bambeke, F. Delafloxacin, a Non-Zwitterionic Fluoroquinolone in Phase III of Clinical Development: Evaluation of Its Pharmacology, Pharmacokinetics, Pharmacodynamics and Clinical Efficacy. *Future Microbiol.* **2015**, *10*, 1111–1123, doi:10.2217/fmb.15.39.
17. Kocsis, B.; Domokos, J.; Szabo, D. Chemical Structure and Pharmacokinetics of Novel Quinolone Agents Represented by Avarofloxacin, Delafloxacin, Finafloxacin, Zabofloxacin and Nemonoxacin. *Ann. Clin. Microbiol. Antimicrob.* **2016**, *15*, 34, doi:10.1186/s12941-016-0150-4.
18. Markham, A. Delafloxacin: First Global Approval. *Drugs* **2017**, *77*, 1481–1486, doi:10.1007/s40265-017-0790-5.
19. Cho, J.C.; Crotty, M.P.; White, B.P.; Worley, M.V. What Is Old Is New Again: Delafloxacin, a Modern Fluoroquinolone. *Pharmacotherapy* **2018**, *38*, 108–121, doi:10.1002/phar.2050.
20. Kaul, G.; Kapoor, E.; Dasgupta, A.; Chopra, S. Delafloxacin Meglumine for the Treatment of Acute Bacterial Skin and Skin Structure Infections (ABSSSI). *Drugs Today (Barc)* **2018**, *54*, 657–666, doi:10.1358/dot.2018.54.11.2878151.
21. Mogle, B.T.; Steele, J.M.; Thomas, S.J.; Bohan, K.H.; Kufel, W.D. Clinical Review of Delafloxacin: A Novel Anionic Fluoroquinolone. *J. Antimicrob. Chemother.* **2018**, *73*, 1439–1451, doi:10.1093/jac/dkx543.
22. Ocheretyaner, E.R.; Park, T.E. Delafloxacin: A Novel Fluoroquinolone with Activity against Methicillin-Resistant Staphylococcus Aureus (MRSA) and Pseudomonas Aeruginosa. *Expert Rev. Anti-Infect. Ther.* **2018**, *16*, 523–530, doi:10.1080/14787210.2018.1489721.
23. Tulkens, P.M.; Van Bambeke, F.; Zinner, S.H. Profile of a Novel Anionic FluoroquinoloneDelafloxacin. *Clin. Infect. Dis.* **2019**, *68*, S213–S222, doi:10.1093/cid/ciy1079.
24. Anwer, M.K.; Iqbal, M.; Muharram, M.M.; Mohammad, M.; Ezzeldin, E.; Aldawsari, M.F.; Alalaiwe, A.; Imam, F. Development of Lipomer Nanoparticles for the Enhancement of Drug Release, Anti-Microbial Activity and Bioavailability of Delafloxacin. *Pharmaceutics* **2020**, *12*, 252, doi:10.3390/pharmaceutics12030252.
25. Scott, L.J. Delafloxacin: A Review in Acute Bacterial Skin and Skin Structure Infections. *Drugs* **2020**, *80*, 1247–1258, doi:10.1007/s40265-020-01358-0.

26. PubChem Delafloxacin Available online: https://pubchem.ncbi.nlm.nih.gov/compound/487101 (accessed on 1 June 2021).
27. Delafloxacin Available online: https://go.drugbank.com/drugs/DB11943 (accessed on 3 June 2021).
28. Higgins, P.G.; Stubbings, W.; Wisplinghoff, H.; Seifert, H. Activity of the Investigational Fluoroquinolone Finafloxacin against Ciprofloxacin-Sensitive and -Resistant Acinetobacter Baumannii Isolates. *Antimicrob Agents Chemother* **2010**, *54*, 1613–1615, doi:10.1128/AAC.01637-09.
29. Lemaire, S.; Van Bambeke, F.; Tulkens, P.M. Activity of Finafloxacin, a Novel Fluoroquinolone with Increased Activity at Acid PH, towards Extracellular and Intracellular , And. *International Journal of Antimicrobial Agents* **2011**, doi:10.1016/j.ijantimicag.2011.03.002.
30. Lee, J.W.; Kim, N.; Nam, R.H.; Kim, J.M.; Park, J.Y.; Lee, S.M.; Kim, J.S.; Lee, D.H.; Jung, H.C. High Efficacy of Finafloxacin on Helicobacter Pylori Isolates at PH 5.0 Compared with That of Other Fluoroquinolones. *Antimicrob Agents Chemother* **2015**, *59*, 7629–7636, doi:10.1128/AAC.01467-15.
31. McKeage, K. Finafloxacin: First Global Approval. *Drugs* **2015**, *75*, 687–693, doi:10.1007/s40265-015-0384-z.
32. Vente, A.; Bentley, C.; Lueckermann, M.; Tambyah, P.; Dalhoff, A. Early Clinical Assessment of the Antimicrobial Activity of Finafloxacin Compared to Ciprofloxacin in Subsets of Microbiologically Characterized Isolates. *Antimicrob. Agents Chemother.* **2018**, *62*, e02325-17, doi:10.1128/AAC.02325-17.
33. Barnes, K.B.; Zumbrun, S.D.; Halasohoris, S.A.; Desai, P.D.; Miller, L.L.; Richards, M.I.; Russell, P.; Bentley, C.; Harding, S.V. Demonstration of the Broad-Spectrum In Vitro Activity of Finafloxacin against Pathogens of Biodefense Interest. *Antimicrob. Agents Chemother.* **2019**, *63*, e01470-19, doi:10.1128/AAC.01470-19.
34. Chalhoub, H.; Harding, S.V.; Tulkens, P.M.; Van Bambeke, F. Influence of PH on the Activity of Finafloxacin against Extracellular and Intracellular Burkholderia Thailandensis, Yersinia Pseudotuberculosis and Francisella Philomiragia and on Its Cellular Pharmacokinetics in THP-1 Monocytes. *Clin. Microbiol. Infect.* **2020**, *26*, doi:10.1016/j.cmi.2019.07.028.
35. Barnes, K.B.; Richards, M.; Laws, T.R.; Nunez, A.; Thwaite, J.E.; Bentley, C.; Harding, S. Finafloxacin Is an Effective Treatment for Inhalational Tularemia and Plague in Mouse Models of Infection. *Antimicrobial Agents and Chemotherapy* **2021**, *65*, e02294-20, doi:10.1128/AAC.02294-20.
36. Bishop, A.J.; Labischinski, H.; Stubbings, W. Finafloxacin HCl - a Novel Experimental Fluoroquinolone with Monotherapeutic Potential for Helicobacter Pylori Eradication. 1.
37. FDA Drug Approval Package Xtoro (Finafloxacin) Otic Suspension Available online: https://www.accessdata.fda.gov/drugsatfda_docs/nda/2014/206307Orig1s000TOC.cfm (accessed on 7 June 2021).
38. PubChem Finafloxacin Available online: https://pubchem.ncbi.nlm.nih.gov/compound/11567473 (accessed on 25 May 2021).
39. Thakare, R.; Singh, S.; Dasgupta, A.; Chopra, S. Lascufloxacin Hydrochloride to Treat Bacterial Infection. *Drugs Today (Barc)* **2020**, *56*, 365–376, doi:10.1358/dot.2020.56.6.3137167.
40. Furuie, H.; Tanioka, S.; Shimizu, K.; Manita, S.; Nishimura, M.; Yoshida, H. Intrapulmonary Pharmacokinetics of Lascufloxacin in Healthy Adult Volunteers. *Antimicrob Agents Chemother* **2018**, *62*, e02169-17, doi:10.1128/AAC.02169-17.
41. Ohya, K.; Takano, J.; Manita, S. In Vitro Mechanistic Study of the Distribution of Lascufloxacin into Epithelial Lining Fluid. *Antimicrobial Agents and Chemotherapy 63*, e02208-18, doi:10.1128/AAC.02208-18.
42. Kishii, R.; Yamaguchi, Y.; Takei, M. In Vitro Activities and Spectrum of the Novel Fluoroquinolone Lascufloxacin (KRP-AM1977). *Antimicrobial Agents and Chemotherapy 61*, e00120-17, doi:10.1128/AAC.00120-17.
43. Tanaka, K.; Vu, H.; Hayashi, M. In Vitro Activities and Spectrum of Lascufloxacin（KRP-AM1977）against Anaerobes. *Journal of Infection and Chemotherapy* **2021**, *27*, 1265–1269, doi:10.1016/j.jiac.2021.03.026.
44. Nenoff, P. Acne Vulgaris and Bacterial Skin Infections: Review of the Topical Quinolone Nadifloxacin. *Expert Review of Dermatology* **2006**, *1*, 643–654, doi:10.1586/17469872.1.5.643.
45. Alba, V.; Urban, E.; Angeles Dominguez, M.; Nagy, E.; Nord, C.-E.; Palacín, C.; Vila, J. In Vitro Activity of Nadifloxacin against Several Gram-Positive Bacteria and Analysis of the Possible Evolution of Resistance after 2 Years of Use in Germany. *Int J Antimicrob Agents* **2009**, *33*, 272–275, doi:10.1016/j.ijantimicag.2008.08.024.
46. Narayanan, V.; Motlekar, S.; Kadhe, G.; Bhagat, S. Efficacy and Safety of Nadifloxacin for Bacterial Skin Infections: Results from Clinical and Post-Marketing Studies. *Dermatol Ther (Heidelb)* **2014**, *4*, 233–248, doi:10.1007/s13555-014-0062-1.
47. Bhagwat, S.S.; Nandanwar, M.; Kansagara, A.; Patel, A.; Takalkar, S.; Chavan, R.; Periasamy, H.; Yeole, R.; Deshpande, P.K.; Bhavsar, S.; et al. Levonadifloxacin, a Novel Broad-Spectrum Anti-MRSA Benzoquinolizine Quinolone Agent: Review of Current Evidence. *Drug Des Devel Ther* **2019**, *13*, 4351–4365, doi:10.2147/DDDT.S229882.
48. Tarek, M.; Elzanfaly, E.S.; Amer, S.M.; Wagdy, H.A. Selective Analysis of Nadifloxacin in Human Plasma Samples Using a Molecularly Imprinted Polymer-Based Solid-Phase Extraction Proceeded by UPLC-DAD Analysis. *Microchemical Journal* **2020**, *158*, 105162, doi:10.1016/j.microc.2020.105162.
49. Liang, W. t; Chen, Y.; Cao, Y.; Liu, X.; Huang, J.; Hu, J.; Zhao, M.; Guo, Q.; Zhang, S.; Wu, X.; et al. Pharmacokinetics and Pharmacodynamics of Nemonoxacin against Streptococcus Pneumoniae in an In Vitro Infection Model. *Antimicrob. Agents Chemother.* **2013**, *57*, 2942–2947, doi:10.1128/AAC.01098-12.
50. Li, Z.; Liu, Y.; Wang, R.; Li, A. Antibacterial Activities of Nemonoxacin against Clinical Isolates of Staphylococcus Aureus: An in Vitro Comparison with Three Fluoroquinolones. *World J. Microbiol. Biotechnol.* **2014**, *30*, 2927–2932, doi:10.1007/s11274-014-1720-2.
51. Poole, R.M. Nemonoxacin: First Global Approval. *Drugs* **2014**, *74*, 1445–1453, doi:10.1007/s40265-014-0270-0.

52. Qin, X.; Huang, H. Review of Nemonoxacin with Special Focus on Clinical Development. *Drug design, development and therapy* **2014**, *8*, 765–74, doi:10.2147/DDDT.S63581.
53. Huang, C.-H.; Lai, C.-C.; Chen, Y.-H.; Hsueh, P.-R. The Potential Role of Nemonoxacin for Treatment of Common Infections. *Expert Opin. Pharmacother.* **2015**, *16*, 263–270, doi:10.1517/14656566.2015.978288.
54. Chang, L.-W.; Hsu, M.-C.; Zhang, Y.-Y. *Nemonoxacin (Taigexyn®): A New Non-Fluorinated Quinolone*; IntechOpen, 2019; ISBN 978-1-78984-473-3.
55. Cheng, S.-L.; Wu, R.-G.; Chuang, Y.-C.; Perng, W.-C.; Tsao, S.-M.; Chang, Y.-T.; Chang, L.-W.; Hsu, M.-C. Integrated Safety Summary of Phase II and III Studies Comparing Oral Nemonoxacin and Levofloxacin in Community-Acquired Pneumonia. *J. Microbiol. Immunol. Infect.* **2019**, *52*, 743–751, doi:10.1016/j.jmii.2018.11.006.
56. Jean, S.-S.; Chang, L.-W.; Hsueh, P.-R. Tentative Clinical Breakpoints and Epidemiological Cut-off Values of Nemonoxacin for Streptococcus Pneumoniae and Staphylococcus Aureus Isolates Associated with Community-Acquired Pneumonia. *J. Glob. Antimicrob. Resist.* **2020**, *23*, 388–393, doi:10.1016/j.jgar.2020.10.017.
57. Yang, J.-J.; Cheng, A.; Tai, H.-M.; Chang, L.-W.; Hsu, M.-C.; Sheng, W.-H. Selected Mutations by Nemonoxacin and Fluoroquinolone Exposure among Relevant Gram-Positive Bacterial Strains in Taiwan. *Microb. Drug Resist.* **2020**, *26*, 110–117, doi:10.1089/mdr.2019.0048.
58. Jones, R.N.; Biedenbach, D.J.; Ambrose, P.G.; Wikler, M.A. Zabofloxacin (DW-224a) Activity against Neisseria Gonorrhoeae Including Quinolone-Resistant Strains. *Diagn Microbiol Infect Dis* **2008**, *62*, 110–112, doi:10.1016/j.diagmicrobio.2008.05.010.
59. Han, H.; Kim, S.E.; Shin, K.-H.; Lim, C.; Lim, K.S.; Yu, K.-S.; Cho, J.-Y. Comparison of Pharmacokinetics between New Quinolone Antibiotics: The Zabofloxacin Hydrochloride Capsule and the Zabofloxacin Aspartate Tablet. *Curr. Med. Res. Opin.* **2013**, *29*, 1349–1355, doi:10.1185/03007995.2013.825591.
60. Rhee, C.K.; Chang, J.H.; Choi, E.G.; Kim, H.K.; Kwon, Y.-S.; Kyung, S.Y.; Lee, J.-H.; Park, M.J.; Yoo, K.H.; Oh, Y.M. Zabofloxacin versus Moxifloxacin in Patients with COPD Exacerbation: A Multicenter, Double-Blind, Double-Dummy, Randomized, Controlled, Phase III, Non-Inferiority Trial. *Int. J. Chronic Obstr. Pulm. Dis.* **2015**, *10*, doi:10.2147/COPD.S90948.
61. Park, H.-S.; Oh, S.-H.; Kim, H.-S.; Choi, D.-R.; Kwak, J.-H. Antimicrobial Activity of Zabofloxacin against Clinically Isolated Streptococcus Pneumoniae. *Molecules* **2016**, *21*, 1562, doi:10.3390/molecules21111562.
62. Mohamed, N.M.; Zakaria, A.S.; Edward, E.A. Antifungal Caspofungin Sensitizes MRSA Isolates Towards Zabofloxacin, A Proteomic Study. *J. Pure Appl. Microbiol.* **2020**, *14*, 559–572, doi:10.22207/JPAM.14.1.58.
